# Efficient and reversible chirality induction between protein and achiral plasmonic assemblies

**DOI:** 10.1038/s41563-026-02586-7

**Published:** 2026-04-15

**Authors:** Ziwei Zhou, Ningwei Sun, Nina Tverdokhleb, Artur Movsesyan, Anja Maria Steiner, Patrick T. Probst, Vaibhav Gupta, Bo Yin, Nicolás Pazos-Peréz, Ramón A. Álvarez-Puebla, Mirjam Taube, Martin Müller, Holger Merlitz, Olga Guskova, Yaroslava G. Yingling, Franziska S. -C. Lissel, Tobias A. F. König, Zhiming Wang, Alexander O. Govorov, Nicholas A. Kotov, Andreas Fery

**Affiliations:** 1 Leibniz-Institut für Polymerforschung Dresden, Dresden, Germany; 2 Technische Universität Dresden, Dresden Center for Computational Materials Science (DCMS), Dresden, Germany; 3 Dresden Center for Computational Materials Science (DCMS), TU Dresden, Dresden, Germany; 4 Institute of Fundamental and Frontier Sciences, University of Electronic Science and Technology of China, Chengdu, China; 5 Aix Marseille University, CNRS, CINAM, AMUtech, Marseille, France; 6 Department of Electrical and Electronic Engineering, Graduate School of Engineering, Kobe University, Kobe, Japan; 7 Synopsys, Canonsburg, PA USA; 8 Department of Physical and Inorganic Chemistry Universitat Rovira i Virgili, Tarragona, Spain; 9 ICREA, Barcelona, Spain; 10 Faculty of Chemistry and Food Chemistry, Technische Universität Dresden, Dresden, Germany; 11 Department of Materials Science and Engineering, North Carolina State University, Raleigh, NC USA; 12 Institute of Applied Polymer Physics (IAPP), Hamburg University of Technology (TUHH), Hamburg, Germany; 13 Department of Physics and Astronomy, Ohio University, Athens, OH USA; 14 Department of Chemical Engineering, University of Michigan, Ann Arbor, MI USA; 15 Biointerfaces Institute, University of Michigan, Ann Arbor, MI USA; 16 Department of Materials Science and Engineering, University of Michigan, Ann Arbor, MI USA; 17 Center for Advancing Electronics Dresden (cfaed), Technische Universität Dresden, Dresden, Germany; 18 Department of Physical Chemistry of Polymeric Materials, Technische Universität Dresden, Dresden, Germany

**Keywords:** Metamaterials, Proteins, Deformation dynamics

## Abstract

Chiral molecules in nature usually show optical activity only in the deep ultraviolet, whereas artificial chiral plasmonic nanostructures can generate much stronger responses at visible and near-infrared wavelengths. An important challenge is whether the abundant biomolecular chirality in nature can be directly transferred to achiral plasmonic systems without elaborate three-dimensional nanofabrication. Here we show that the mechanical stretching of protein molecules anchored within achiral gold nanoparticle assemblies strongly enhances and reversibly modulates plasmon-coupled circular dichroism. Stretching amplifies the chiroptical response to an ellipticity of 1.18° and a dissymmetry factor of 0.2, far exceeding conventional hotspot-based strategies. Repeated stretching and relaxation further enable reversible switching over more than 100 cycles. Simulations and in situ spectroscopy indicate that the deformation of protein changes its conformation and dipole alignment, thereby strengthening the plasmonic chiral response. These findings establish a route to achieve dynamically controllable chiroptical activity in achiral plasmonic assemblies, revealing how small biomolecular deformations can strongly influence plasmonic responses of much larger nanostructures.

## Abstract (web summary)

Mechanical modulation of protein conformation enables the reversible induction of strong plasmon-coupled circular dichroism in otherwise achiral plasmonic assemblies.

## Main

The chirality of nanostructures results in intense circular dichroism (CD) when the nanomaterial exhibits high polarizability and a size comparable with the wavelength of light^1^. The chiroptical effects in this case are several orders of magnitude stronger than those of organic materials with low polarizability, extending the range of observable CD peaks from the ultraviolet (UV) to the near-infrared (NIR) range, which is not typical for chiral molecules. Chiral nanostructures from plasmonic nanomaterials feature particularly strong chiroptical activity and active polarization control^2–6^ via their variable nanoscale geometries. For example, the assemblies of plasmonic nanoparticles (NPs) can be configured into predesigned mirror-asymmetric shapes, such as tetrahedra^7^, helices^8–12^ or scissors^13–17^, which stimulated a wide range of applications^18,19^.

There has also been a growing effort to better understand the multiple mechanisms by which biomolecules induce chirality in plasmonic nanostructures^5,8,9,20^. Although state-of-the-art plasmonic nanostructures sometimes reach giant chiroptical activity, they typically possess permanent chiral shapes with time-invariable mirror asymmetry^4,5,8,9^. Achieving reversible induction of chirality and retaining high CD amplitudes would be a fundamental and technological milestone in this field, as it could offer (1) insights in the trans-scale propagation of mirror asymmetry and (2) a pathway for strong polarization rotation with real-time tunability of magnitude and spectral position.

Plasmon-coupled circular dichroism (PCCD; also termed as molecule-induced plasmonic circular dichroism) presents an opportunity for the reversible induction of chirality by converting the chirality of molecules into the chirality of electron gas in metal nanostructures without altering the overall shape of the latter^8,21–29^. In other words, a typical biomolecule induces a chiral response in a nearby NP via electrostatic interaction rather than serving as a shape-defining template. However, the efficiency of PCCD remains low due to the size mismatch between molecules and nanostructures and the fast averaging of chiral configurations. Moreover, its strength rapidly decreases the molecule–nanostructure distance, as the localized plasmonic near field decays exponentially away from the metal surface^25^. To address these challenges, common strategies for enhancing PCCD included designing nanogaps that position chiral molecules within strong electric field hotspots. Even so, the resulting PCCD amplitudes and *g*-factors remain 10–100 times smaller than those observed in plasmonic nanostructures with permanent structural chirality. This disparity urges the development of alternative, more effective methods to further enhance the PCCD intensity.

It is reasonable to infer that investigating the role of chiral molecules in the context of PCCD could serve as an effective alternative, as chiral molecules represent the fundamental origin of chirality in PCCD. However, studies focusing on the molecular aspect of PCCD remain limited^8,25^, primarily due to challenges in tracking the reconfiguration of individual biomolecules and monitoring the resulting changes in PCCD. Addressing these limitations and optimizing molecular contributions to PCCD could potentially lead to a substantial amplification of chiroplasmonic activity.

On the other hand, the stimulus-responsive reconfiguration of structurally chiral nanostructures and related chiroplasmonic effects are also exciting from both fundamental and technological perspectives. They have been demonstrated at much larger scales^30^—for instance, through the mechanical deformation of chiral nanocomposites and kirigami—the potential for achieving the strong cyclic modulation of the electrostatic coupling mechanism underlying PCCD remains largely unexplored^8,25^. A deeper investigation into the molecular contributions to PCCD could provide valuable insights for overcoming this challenge.

## Design and assembly of linear NP–protein complex array with pure PCCD

To realize the reversible and tunable induction of chirality from biomolecules to plasmonic states, we selected spherical NPs and assembled them into a densely packed one-dimensional (1D) chain by using template-assisted colloidal self-assembly^12,13,31,32^. The NPs, with an average diameter of 70.6 nm, were conjugated to bovine serum albumin (BSA) (Supplementary Fig. 1). BSA molecules anchor to the gold surface primarily through the unique free thiol at Cys34, located in domain I-A near the protein surface. All other cysteines in BSA form 17 intramolecular disulfide bridges, leaving only Cys34 available for covalent binding. The crystallographic characterization of gold–albumin complexes has directly shown Au–S coordination at this residue^33^, and tandem mass spectrometry confirms the absence of other free thiols^34^. This assignment is consistent with spectroscopic analyses locating Cys34 at an exposed position on the protein surface^35^. Hence, the Au–S bond at Cys34 serves as the dominant anchoring point that links the protein shell to the NP surface.

**Fig. 1:**
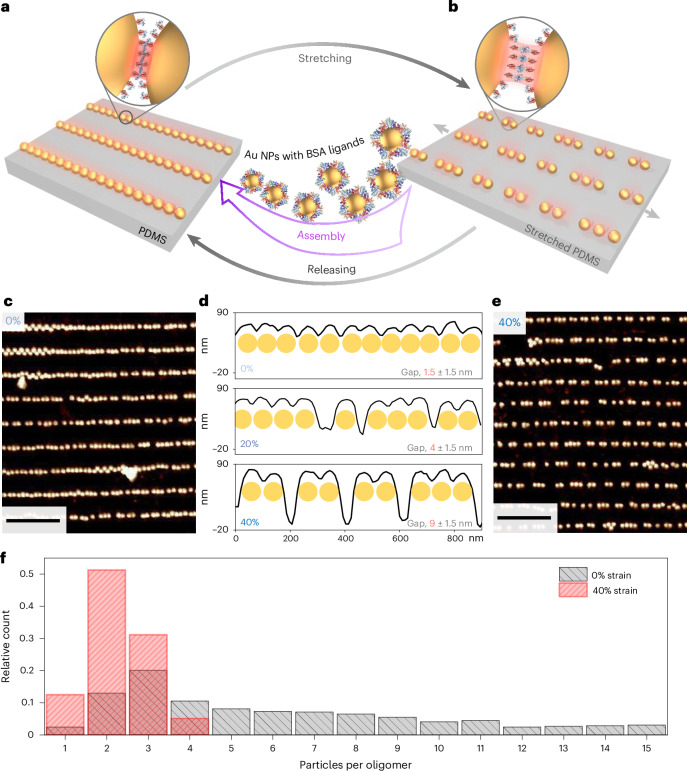
Stretchable 1D BSA-coated Au NP array is designed to obtain robust pure PCCD and to explore the role of molecular conformation in it by deforming the substrate. **a**, Schematic of the 1D BSA-coated Au NP array. Each BSA molecule is located in the plasmonic hotspot between adjacent NPs. All of them contribute to PCCD. The centimetre-square assembly has plasmonic hotspots (visualized by the red clouds between the NPs), enhancing the chiroptical effects. **b**–**e**, Soft PDMS substrate is stretched along the chain direction (**b**), gradually enlarging the gaps between the NPs in each chain (**c**–**e** show the AFM images. Scale bar, 500 nm), which causes the BSA molecules in the hotspots to transform from a highly squeezed state into a highly stretched state, allowing us to monitor the BSA conformational changes in real time. **f**, Oligomer length distributions of the original state and the 40% strain state, showing the splitting of the BSA-coated Au NP chains during stretching. Source data

Before the formation of the 1D array, the isolated NPs coated with BSA ligands displayed negligible PCCD^26,36,37^ (Supplementary Fig. 2b). Note that linear assemblies of predominantly spheroidal NPs are achiral at the nanoscale, enabling us to observe the effect of the time-variable mirror-asymmetric shape of BSA on the PCCD of NP chains (Fig. 1a and Supplementary Fig. 2). The inter-NP gaps in the chains are smaller than the thickness of the isolated BSA-coated gold NPs (Au NPs) due to the strong attraction between the NPs during the drying process (Supplementary Fig. 1), resulting in the relatively soft BSA shells being squeezed and intertwined^38^. To ensure the uniform deformation of NP chains during stretching, a thin adhesive layer of polyethyleneimine (PEI) was cast onto the polydimethylsiloxane (PDMS) substrate (Fig. 1b). The NPs are, therefore, partially embedded by ~20 nm in the PEI layer^31^, allowing the PEI layer to act as a matrix that transfers the strain from the PDMS substrate to the NP chains and subsequently to the BSA molecules between the NPs (Supplementary Video 1). Stretching or relaxation of PDMS was performed with a custom-built device (Supplementary Fig. 3), which completes either motion within a few seconds. With increasing strain, atomic force microscopy (AFM) images indicated that the NPs were gradually separated (Fig. 1c–e and Supplementary Fig. 4). Simultaneously, the tangled proteins between adjacent NPs also bear tension, enabling them to reconfigure from their pristine state into more elongated states. This system provides a model that allows us to access a range of protein conformations, and systematically investigate the induction of chirality from biomolecules to plasmons.

## Reversible PCCD and its anomalies

**Fig. 2:**
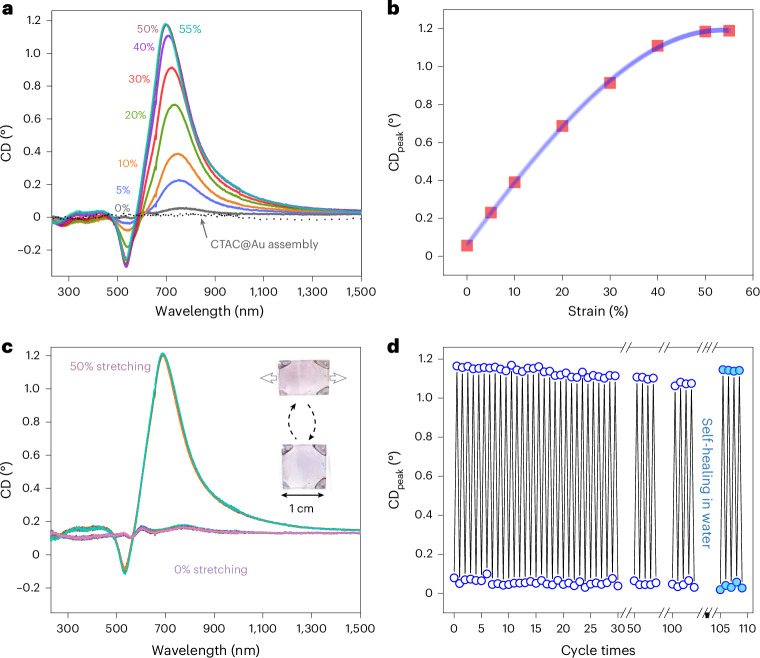
Strain-induced PCCD enhancement of BSA-coated Au NP 1D assemblies on continuous substrate stretching. **a**, PCCD spectra of BSA-coated Au 1D assembly array with external strain increasing from 0% to 55%. The black dotted line depicts the CD spectrum of a CTAC-coated Au NP assembly, showing no CD signal due to the achirality of the CTAC molecules. **b**, Red squares and purple line plot changes in the peak PCCD values during the stretching–relaxation process. This increasing trend fits a half-linear curve (dotted line). **c**, Collective PCCD spectra of 15 stretching–relaxation cycles, showing the outstanding reproducibility of the response. The insets show the optical photographs of the 1D chiral assembly array in the stretching and relaxation states. **d**, Records of the peak CD values during the cycling test over 100 cycles and the self-healing behaviour of the CD value after immersing into water overnight. Source data

The plasmonic hotspots in the 1D NP assembly promote collective PCCD that can be detected using common spectroscopy techniques (~50 mdeg (Fig. 2a (dark grey line)), with ellipsometry used here; Supplementary Note 1). This PCCD observed in the visible–NIR range shows an opposite spectral sign compared with the molecular chirality of BSA, which is located in the UV region (Supplementary Fig. 5 shows the full CD spectrum; analysing all the CD spectra presented a contribution of CD scattering must also be considered). In stark contrast, assemblies using achiral molecules as ligands (for example, hexadecyltrimethylammonium chloride (CTAC)) show no detectable CD at the plasmon resonance. Also, on stretching, no discernible CD signal was observed, consistent with the non-chiral nature of CTAC molecules and NP chains (Supplementary Fig. 2d).

When applying uniaxial strain of 0%–55% to the elastic substrate along the chain direction, the NP chains at the surface gradually split into oligomers and the interparticle gaps gradually widened (Fig. 1c–e and Supplementary Fig. 4). According to existing theories, an increase in the centre-to-centre distance of the NP–molecule complex should lead to a rapid decrease in PCCD as the plasmonic hotspots weaken^21–25,28,36,37^. Contrary to this expectation, PCCD increased drastically by a factor of 22 when the nanogaps were enlarged by stretching (Fig. 2a,b). The PCCD intensity reached up to 1.18° at a wavelength of ~700 nm with a *g*-factor of 0.2 (Fig. 2a and Supplementary Fig. 6). Both CD amplitudes and *g*-factor increased by 10 and 1,000 times, respectively, compared with previously reported PCCD values (Extended Data Table 1). The achieved CD amplitudes and *g*-factors are comparable with those of conventional structural CD, thereby redefining the efficiency limits traditionally associated with PCCD.

The PCCD of our NP chains exhibited high sensitivity to external strain applied along the chain direction. The strain-induced PCCD increase displayed distinct nonlinear behaviour; following an initial linear increase, there was a gradual slowdown starting from approximately 25% strain, eventually reaching a plateau around 55% strain (Fig. 2b). On relaxation, the system reverts to its initial low PCCD value.

The stretching process enables continuous and gradual control over PCCD, demonstrating high repeatability. Even after 100 cycles of stretching between 0% and 50% strain (Fig. 2d), the high modulation contrast was maintained, with only a slight reduction (4%) in CD intensity. The CD amplitude was restored to its original state when the sample was immersed in water overnight, allowing the stretched protein segments to refold. However, when the strain exceeded 50%, PCCD began to decrease, accompanied by a gradual blueshift in the longitudinal plasmonic peak, indicating a reduction in NP–NP coupling (Supplementary Fig. 7). Overstretching also resulted in a decrease in CD due to the detachment of highly strained BSA molecules.

## Validation of PCCD measurements

As benchmarks for attributing the spectroscopic data, the CD amplitude of the stretched BSA-coated Au NP chains changed little when the sample was rotated about the normal to the film surface and the photon propagation vector (Supplementary Fig. 8), effectively ruling out the contribution of linear dichroism in the PCCD. In addition to ellipsometry, conventional CD spectrometry was utilized to assess the PCCD during stretching (Supplementary Fig. 9), and it also exhibited the same increase in PCCD and CD amplitudes.

Moreover, to further validate the observed effect as a consequence of strain-enhanced PCCD, NP chains were assembled using smaller and larger NPs (with diameters of 50 nm and 90 nm, respectively), following the same assembly process (Supplementary Figs. 10 and 11). Both sizes showed stretching-induced rise and decrease in PCCD, thereby excluding potential artifacts specific to NP preparation.

## Monitoring of plasmonic properties of NP–protein complexes during stretching

**Fig. 3:**
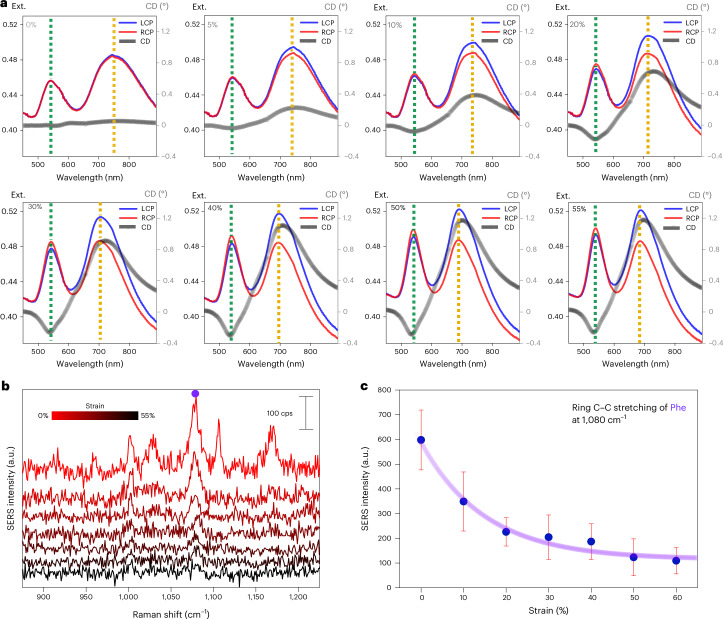
Spectroscopic data for the protein-coated NP chains during the strain-induced PCCD modulation. **a**, Extinction (Ext.) spectra of the BSA-coated Au NP chain assembly on a PDMS substrate with increasing strain from 0% to 55% for incident LCP and RCP light. The green dashed lines label the transversal plasmonic mode and the orange dotted lines indicate the longitudinal plasmonic mode. The grey lines represent the corresponding CD spectra for a comparison. **b**, SERS intensities of the 1D assembly during stretching. A 785-nm laser line was used with a power of 1 μW at the sample and acquisition time of 1 s. **c**, Intensity of the SERS spectra as a function of strain for the ring C–C stretching band of Phe at 1,080 cm^−1^. Each point represents the mean of *n* = 10 technical replicates (SERS acquisitions collected at distinct positions on the same NP chain assembly/substrate at each strain). Error bars indicate ±standard deviation across the measurements. Source data

The optical mechanisms behind the cyclic modulation of PCCD can be elucidated from the spectroscopic data presented in Fig. 3a. In the circularly polarized extinction spectra of the PDMS-supported NP assembly (Supplementary Fig. 6a shows the extinction spectrum corrected by subtracting the PDMS substrate contribution), two main plasmonic peaks were observed at 540 nm and 740 nm, marked by the green and orange dotted lines, respectively. The position of the transversal mode (green dotted lines) remains nearly constant during stretching. However, as the NP chain begins to split on exceeding the strain disruption limit, the longitudinal mode (orange dotted lines) experiences a blueshift. Simultaneously, the sharpening of both plasmonic peaks occurs as the length distributions of NP oligomers become increasingly centralized during stretching (Fig. 1f).

The difference in extinction between left-handed circularly polarized (LCP) light and right-handed circularly polarized (RCP) light primarily arises from the 740-nm longitudinal mode^36,37^, suggesting that the chiral electronic states of BSA positioned between NPs predominantly couple into the longitudinal plasmonic mode. Due to the strong delocalization, the extinction peaks for LCP and RCP are nearly identical in the spectral positions, contrasting with the conventional Cotton effect seen in highly localized excitonic states. In particular, the difference in sign arises from the electrostatic nature of PCCD and the achiral character of the nanostructures. In fact, the appearance of anti-Cotton CD peaks serves as direct means to distinguish PCCD from structural CD, as they can often be easily confused^36,37^.

The phenomenon of plasmonic hotspots decaying due to increased particle spacing during stretching can be clearly illustrated through surface-enhanced Raman scattering (SERS) analysis (Fig. 3b,c). In its unstretched state, the NP-BSA chains exhibit discernible spectral features associated with phenylalanine (Phe) at 1,004, 1,030 and 1,080 cm^−1^, along with fainter signals at 914, 955, 1,100 and 1,168 cm^−1^, attributed to lysine (Lys)^39^. Both contributions are pronounced due to the high proportion of these two amino acids in BSA. Two distinct Raman scattering phenomena manifest during stretching: (1) the disappearance of Lys signature peaks and (2) a reduction in the absolute intensity of Phe peaks. These effects can be rationalized by the strain-induced expansion of the inter-NP gap dimensions (Fig. 1d), leading to a subsequent decrease in the SERS intensity (Fig. 3c)^40^. Conversely, changes in the relative intensity of Phe signals distinctly indicate their reorientation in response to protein unfolding^41^, serving as a clear illustration of the surface selection rules^42^. When the substrate is relaxed, the SERS spectra exhibit a reorganization of the protein’s initial configuration (Supplementary Fig. 12), confirming the strain-induced reconfiguration of BSA.

Although local plasmonic near fields exist within the nanogaps and may transiently bias the orientation of adsorbing proteins, this effect is minor in our system. During the solution-to-dry assembly, capillary compression rapidly dehydrates and collapses the BSA layer, and in the subsequent dry-state stretching stage, the nanogaps widen and the field strength decays. Therefore, the observed alignment and chiroptical response are dominated by mechanical deformation more than by electric-field-driven orientation.

## Reversible chirality in achiral nanostructure via biomolecular deformation

**Fig. 4:**
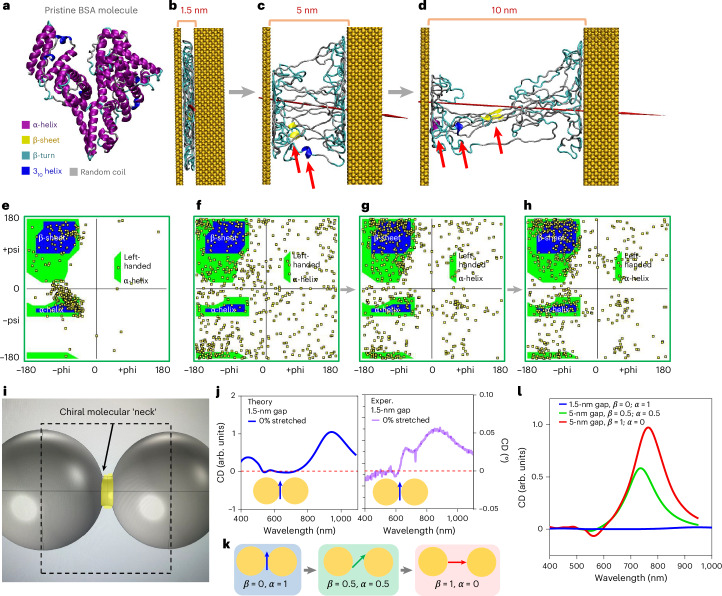
All-atom protein simulation of the deformed BSA protein during stretching and PCCD computation based on molecular simulation. **a**, Pristine BSA molecule that presents a globular shape, containing abundant α-helices as the Ramachandran map shown in **e**, where *φ* (phi) and *ψ* (psi) denote the backbone dihedral angles of the protein. **b**, Squeezed BSA between two gold walls with a gap size of 1.5 nm to simulate BSA in the pristine nanochain assembly that is highly squeezed by capillary force. Here the majority of regular secondary structure is absent; meanwhile, more β-turns and random coils formed, as shown in **f**. **c**,**d**, Stretching induces the recovery of some of the regular secondary structures in the protein, as indicated by the small red arrows, as well as shown by the Ramachandran maps in **g** and **h**. The changes in the protein molecular dipole during stretching are shown by the deep red lines. Both deep red lines were scaled down with factors of 0.18 in **c** and 0.12 in **d**, as their original lengths would occupy too much space in the figure. **i**, Schematic of the NP–protein assembly used in the PCCD calculations, modelled in COMSOL with a chiral continuous-medium approximation. **j**, Comparison of theoretical (left) and experimental (right) CD spectra for a 1.5-nm gap without stretching, showing weak CD bands at the longitudinal and transverse plasmon resonances due to plasmon–chiral medium interactions. **k**, Model of chiral molecules in the gap, represented as a mixture of stretched (*β* = 1, *α* = 0) and non-stretched (*β* = 0, *α* = 1) conformations, with intermediate cases included. **l**, PCCD calculations for different gap sizes and dipole orientations, showing that stretched molecules yield stronger CD signals, in agreement with experimental observations. Source data

To unravel the effect of BSA conformation on CD spectra, a closer examination of the protein’s structural configuration is necessary. As the BSA-coated Au NPs congregated during assembly, capillary forces compelled the NPs to progressively draw closer together. This results in close-packed assemblies (Fig. 1c,d and Supplementary Fig. 1). Consequently, the segments of adjoining BSA shells became intertwined^38^. During the drying process, BSA molecules within these confined crevices experienced substantial compression; therefore, their conformation after drying resembles a ‘pancake’ configuration, as illustrated by the full-atomistic molecular dynamics (MD) simulation of the protein (Fig. 4b). A pristine BSA molecule is known to be abundant in α-helices, accompanied by the presence of non-regular β-turns (Fig. 4a,e). The calculated CD spectrum of a pristine BSA revealed typical peaks of an α-helix (Supplementary Fig. 13). The most probable anchoring site for the covalent attachment of BSA to Au NPs is the free thiol of Cys34, which resides in the sub-domain I-A of domain I of the protein. All other sulfur atoms form disulfide bridges and are largely buried within the albumin scaffold, rendering them inaccessible for direct Au–S bonding^43^. Consequently, in our MD simulations, we stabilized the BSA corona on the NP surface primarily through the Cys34 anchoring point, whereas the remainder of the protein interacts via non-covalent electrostatic and hydrophobic contacts.

When the protein is squeezed between the NPs, a large portion of the comparatively larger α-helices undergoes substantial disruption (Fig. 4b,f). Concurrently, the occurrence of random coils and smaller-volume non-regular secondary structures, such as β-turns, increases. Hence, the disruption of the secondary structure corresponds to a reduction in the protein’s chirality. The calculated CD spectrum based on the squeezed model of BSA also validated this point (Supplementary Fig. 13), where the dips around 210–220 nm (representing α-helix) nearly disappeared, whereas the main dip appeared between 200 and 210 nm, reflecting the predominance of random coils and β-turns. It is noteworthy that in the compressed state, the pancake-shaped protein aligns its molecular dipole parallel to the gold surface (Fig. 4b, deep red lines), which is the least favourable orientation for inducing PCCD^22^. Consequently, the reduction in molecular chirality, coupled with this unfavourable dipole alignment, leads to the subdued PCCD observed in the unstretched NP assembly. However, stretching the nanochain assembly creates additional space for the recovery of squeezed secondary structures. As a result, we observe a partial revival of α-helices alongside the emergence of β-sheets during stretching (Fig. 4c,d,gh). The calculated CD spectra of the stretched BSA molecules (Supplementary Fig. 13) clearly reveal the recovery of the CD dip around 225 nm (α-helix), and a weakening of the dip around 200 nm (random coils), indicating that stretching facilitates the transformation of some random coils and short β-sheet turns into α-helices. This revival also implies a recovery in molecular chirality. Simultaneously, stretching strongly prompts the molecular dipole to align with the nanochain direction, which is the most conducive orientation for PCCD^22^.

We monitored residue reorientation using Phe and Lys as representative markers. Under in situ SERS during stretching (Fig. 3b,c), the Phe ring C–C stretching band at ~1,080 cm^−1^ exhibits the strongest response among aromatic residues, enabling reliable orientation tracking. Lys provides a complementary probe owing to its clear SERS features and high, broadly distributed abundance in BSA, thereby reporting global conformational changes. Consistent with this selection, dihedral-angle distributions for Phe and Lys (Supplementary Fig. 14 and Supplementary Note 3) evolve from narrower, surface-parallel orientations at 1.5 nm to broader angles at 10 nm, supporting a stretching-induced transition of BSA from parallel to more normal orientation relative to the gold surface. This alignment is accompanied by a pronounced increase in the magnitude of the dipole strength (Fig. 4d and Supplementary Fig. 15; Supplementary Video 2 shows a comprehensive video).

In summary, the restoration of molecular chirality, coupled with intensified molecular dipole alignment along the favourable direction, collectively contributes to the unexpected surge in PCCD during stretching. To substantiate the protein simulations, electromagnetic PCCD computations^22,44^ were conducted to directly confirm the stretching-induced increase in CD intensity (Fig. 4i–l and Supplementary Note 4). The results validate that the stretched chiral molecule leads to a substantial improvement in PCCD performance, in good agreement with the CD measurements.

To experimentally validate the MD simulation, the attenuated total reflectance (infrared) technique was used to monitor the structural changes in BSA within the nanochain assembly during stretching (Supplementary Fig. 16). Initially, the broad amide I band, centred around 1,660 cm^−1^, indicated a substantial contribution from both random coils and β-turns in the protein structure^45–47^. On stretching, the amide I maximum shifted slightly to 1,655 cm^−1^, clearly within the range of 1,657–1,648 cm^−1^, characteristic of α-helical structures. Additionally, a shoulder peak (Supplementary Fig. 16, light green region) appeared between 1,630 and 1,610 cm^−1^, within the range of 1,641–1,609 cm^−1^, corresponding to β-sheet structures^45,48^. These observations suggest that stretching induces the increased formation of ordered secondary structures, specifically α-helices and β-sheets. In particular, these infrared measurements are in close agreement with the results of protein simulations.

Electromagnetic modelling was done for the model (Fig. 4i). The model computed using COMSOL (Supplementary Note 4) strikingly reproduced the experimental spectral shapes of CD and the major tendencies with the stretching. In our COMSOL theory, we observe how a typical CD band appears with a minimum at ~530 nm (collective transversal plasmons) and a maximum at ~650 nm (collective longitudinal plasmons). The combination of experiments and simulations has enhanced our comprehension of the PCCD mechanism at the molecular level. This integration has facilitated a straightforward PCCD switching process through the manipulation of stretched and relaxed molecular states.

**Fig. 5:**
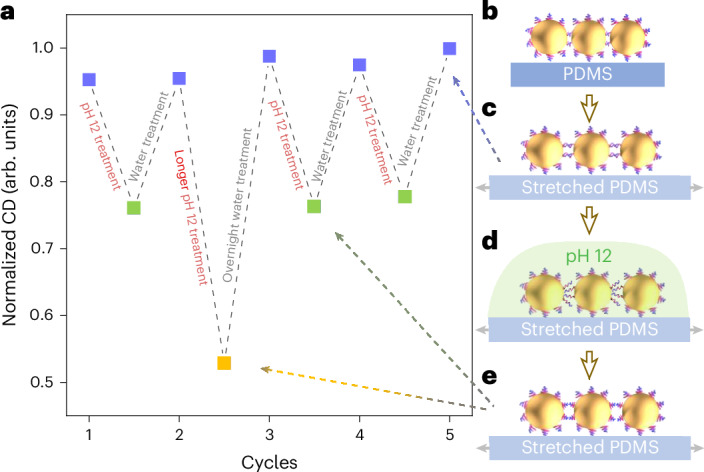
pH-induced PCCD switch of the BSA-coated Au NP array. **a**, Switchable PCCD spectra based on the conformational change of BSA under alkali and neutral treatments. **b**, Unstretched BSA-coated Au NP array with the BSA molecules positioned between adjacent NPs being intertwined with each other. **c**, On stretching the PDMS substrate, the Au NPs drifted apart, leaving the intertwined BSA molecules stretched. Furthermore, the stretched BSA molecules would induce highly elevated PCCD, as indicated by the blue squares in **a**. **d**, Holding this stretched state and putting the assembly into a sodium hydroxide (pH 12) solution would break the hydrogen bonds and unfold the protein, which would make the molecular segments freely stretch in the alkali solution. **e**, Holding this stretched state and drying the assembly from the alkali solution, the molecular segments would spontaneously adhere to their respective Au NPs instead of becoming intertwined with neighbouring BSA segments, as the gaps between adjacent NPs are too wide now. In this case, the less-stretched BSA molecules would lead to a conspicuous decrease in the CD signal (as evident from the green squares in **a**). Interestingly, relaxing and submerging the alkali-treated assembly into water would cause the BSA segments to reconfigure and intertwine once again. Subsequent drying and stretching of the assembly would contribute to a restoration of the CD signal to its original level (indicated by the blue squares in **a**). Source data

We used pH changes to validate the effect of BSA tangling (Fig. 5). As previously shown, when we stretched the chain assembly, markedly elevated CD values were observed (Fig. 5, blue squares) because of the stretching of the entangled BSA molecules located between adjacent particles. Subsequently, we immersed the stretched assemblies into a sodium hydroxide solution (pH 12) for 2 h, which is known to induce a sustainable conformational expansion of BSA by disrupting intramolecular hydrogen bonds and exposing previously buried segments^49^. This treatment, thus, allowed BSA chains to unfold more fully and facilitated the separation of initially entangled protein molecules. Consequently, when drying occurs under a stretched configuration, the distance between adjacent NPs remains sufficiently large such that the intervening protein molecules are too far apart to engage in entanglement. As water evaporates, these protein chains are constrained to adsorb individually onto the surfaces of their respective NPs, rather than forming an interconnected network. This resulted in a looser NP arrangement, due to the absence of BSA-mediated entanglement between particles.

When stretching the relatively ‘looser’ alkali-treated assembly, the BSA molecules positioned between the adjacent NPs were no longer subjected to the same degree of stretching as before. This reduction in stretching of these BSA molecules led to a conspicuous decrease in the CD signal (Fig. 5, green squares). Interestingly, this decreased CD can be recovered. The stretched, alkali-treated assembly was immersed in water for 2 h to relax the BSA segments. During drying, we gradually relaxed the strain on the PDMS substrate; the BSA segments can gradually touch the neighbouring BSA segments, and got reorganized and re-entangled with the evaporation of water. These previously unfolded segments self-associated into oligomeric or fibril-like networks—a phenomenon widely observed in protein film formation and dry-state aggregation^38^. Subsequent stretching of the assembly restored the CD signal to its original level (Fig. 5, blue squares). This process demonstrated high reversibility and can be conveniently controlled by alternating between alkali and water treatments. Furthermore, when the assembly was immersed in a pH 12 solution overnight, the CD value experienced a sharp decline (Fig. 5, orange square). A longer alkali treatment time led to a more complete disentanglement of the BSA molecules. However, after an overnight water treatment, the CD signal was again reinstated. This experimental evaluation conclusively validates the pivotal role of intermolecular entanglement in determining the PCCD signal intensity.

To directly probe the role of BSA in generating the PCCD response, we performed proteolytic digestion using Proteinase K (ref. ^50^). The Au NP assembly supported on PDMS was kept at ~50% strain to allow sufficient interparticle spacing for enzymatic access. A 100 µg ml^−1^ of Proteinase K buffer solution was applied and incubated at 50 °C for 1 h. As shown in Supplementary Fig. 17, the original PCCD signal (black) obviously decreased after 1 h of digestion (red) and almost vanished after overnight treatment (purple), indicating the removal of the BSA corona. Importantly, the plasmonic extinction spectra remained largely preserved, and AFM imaging confirmed that the NP lattice survived the digestion process. Together, these results provide direct evidence that the chiroptical activity originates predominantly from the presence of surface-bound biomolecules, whose removal leads to the loss of optical chirality.

## Outlook

The efficient and reversible induction of chirality based on electromagnetic interactions between molecular and plasmonic states in nanostructures surpasses the limitations of thermodynamics and the high-energy barriers typically associated with structural chirality. The enhanced CD spectra and elevated *g*-factors challenge the conventional notion of weak chiroptical responses in PCCD. The electromagnetic interactions between biomolecules and achiral NPs enables the dynamic reversal of chirality. Moreover, this reconfiguration can be alternatively triggered by a variety of stimuli, including pH, solvents, light, electric fields and temperature, thereby providing versatility for tailoring diverse CD devices without the need for complex 3D nanofabrication processes. The strong effect of protein stretching on PCCD deepens our comprehension of chirality induction mechanisms and conformational changes in proteins. Our findings also provide an avenue to technologically relevant NP assemblies with field-induced reversibly tunable chiroptical activity.

## Methods

### Chemicals

Hydrogen tetrachloroaurate (>99.9%), sodium borohydride (99%), L-ascorbic acid (>99%), BSA (98%), CTAC (25 wt% in water), PEI (*M*_w_, 2 kg mol^−1^, linear, 50 wt% in water), Proteinase K and Trizma base were purchased from Sigma-Aldrich. Hexadecyltrimethylammonium bromide (99%) was supplied by Merck. Sodium hydroxide (1-M) solution and trisodium citrate (>99%) were received from Grussing. SYLGARD 184 PDMS elastomer kits were purchased from Dow Corning. All chemicals were used without further purification. High-purity deionized water (18.2 MΩ cm^−1^) was used in all aqueous preparations.

### Synthesis and functionalization of single-crystalline NPs

The Au NPs were synthesized as previously reported, followed by a ligand exchange process to coat them with BSA^31^. First, 2-nm Au seeds were prepared through the reduction of hydrogen tetrachloroaurate using sodium borohydride with hexadecyltrimethylammonium bromide as a stabilizer. Further, the Au seeds were grown twice in a solution (containing hydrogen tetrachloroaurate, ascorbic acid and CTAC) to a final diameter of ~70.6 ± 1.2 nm. Additionally, in the last growth step, a syringe pump system was used to ensure the kinetic control of Au growth. The final product was collected by centrifugation and washed twice with a 2-mM CTAC solution. Finally, the CTAC stabilizers could be readily exchanged with chiral ligands such as BSA^31^.

### Large-scale 1D NP assembly

The wrinkled templates with a wavelength and amplitude of ~370 nm and ~35 nm, respectively, were obtained according to a previously published procedure^31^. PDMS was prepared by casting the mixed crosslinker/prepolymer mixture (1:5, SYLGARD 184, Dow Corning) in a levelled polystyrene dish and then by degassing in a vacuum. The PDMS mixture was crosslinked at 80 °C with a final thickness of ~2 mm. The cured PDMS was cut into 1 × 4.5-cm^2^ strips. To achieve PDMS wrinkles, these strips were fixed in a custom-built stretching device and elongated by 40%. The elongated PDMS strips were then O_2_-plasma-treated (Flecto 10, Plasma Technology) for 120 s (100 W, 0.3 mbar of O_2_). The plasma-treated PDMS strips were then cooled to room temperature and slowly relaxed. The obtained PDMS wrinkling was cut into 1 × 1-cm^2^ strips as templates to guide the NPs into closely packed 1D linear assemblies by spin coating^31^. Three microlitres of Au NP suspension ([Au^0^] = 12 mg ml^−1^, pH 11) was spread onto the PDMS wrinkled template, followed by a two-stage spin-coating process (30 s at 1,500 rpm, and 30 s at 4,000 rpm, photoresist spinner, Headway Research). After drying, the assembly took on a rose/grey colour with angle-dependent anisotropy.

The 1D NP assemblies trapped inside the PDMS wrinkles were then wet transferred onto a flat PDMS substrate for better optical performance. The target PDMS with a crosslinker/prepolymer mixing ratio of 1:15 was cured as above. Subsequently, the target PDMS substrate was incubated with 10 mg ml^−1^ of PEI solution for 3 h to apply an adhesion layer on top, promoting the complete transfer of NP assemblies. For the wet transfer, a droplet of water (pH 9) was placed on the centre of the target PDMS substrate. With a pressure of 100 kPa, the NP-filled PDMS stamp was pressed onto the target PDMS. After drying and detaching, the 1D NP chains were transferred to the flat PDMS substrate. A mild annealing step was used to facilitate the intermolecular entanglement of BSA within the assembly.

### Protein simulations

Full-atomistic MD simulation of protein between two Au NPs was carried out using the Nanoscale Molecular Dynamics (v.2.0) software^51^ with the all-atom optimized potential for liquid simulations force field. The structure of pristine BSA molecule was downloaded from the Protein Data Bank library (PDB 3V03). Au NPs were approximated using two bulk gold walls considering the obvious protein/NP size ratio. The walls were built using the plug-in of Inorganic Builder of visual molecular dynamics software^52^. Taking the size of a face-centred-cubic gold unit cell as 4.08 × 4.08 × 4.08 Å, the dimensions of the walls were set as 1 × 50 × 50 (a fixed wall) and 5 × 50 × 50 (a mobile wall) unit cells, respectively. The van der Waals parameters for gold atoms were modelled according to ref. ^31^. To mimic the capillary forces bringing the NPs into a linear assembly in experiments, as well to model the stretching process, a steered MD simulation^53^ method was used to apply a force on all the atoms of a mobile gold wall. The time step of simulations was 0.25 fs.

In line with the experimental setup, the initial stage of the simulations represented an adsorption of BSA onto a fixed gold wall immersed in TIP3P water (*NPT* ensemble, Nose–Hoover thermostat, *T* = 298 K, *P* = 1 atm). The prime solvation of the BSA–gold system was performed using visual molecular dynamics software, where water molecules within 3.4 Å from the protein, NP and ions were removed to avoid steric clashes. To ensure the electroneutrality of the simulation box, 16 Na^+^ ions were added to the simulation system, since at neutral pH, the BSA’s net charge corresponds to –16e^−^. Then, 16 sodium and 16 chloride ions were placed into the simulation box as charge-balancing counterions. The electrostatic interactions were calculated using particle mesh Ewald method.

The adsorption of the protein onto the gold wall immersed in TIP3P water required 60 ns, following an equilibration period of a 20-ns MD run. Alongside the simulations of explicit water, the BSA adsorption was studied in implicit water modelled as a dielectric background with a permittivity of 80 using a generalized Born implicit solvent model, and in vacuum. For each system, the resulting conformation of the protein was analysed with a special emphasis on the secondary structure elements. We note here that independent of the water model applied, the modelling showed similar trends in changing the BSA conformation and the reorientation of its dipole moment. Specifically, the regular secondary structures of the BSA, such as α-helices, partially transformed to unstructured coils and β-turns during adsorption on the gold surface. The type of environment (vacuum, implicit water or explicit water) mainly influenced the extent of helix disruption. For all the water models, during the adsorption process, the dipole moment of the protein tends to align parallel to the gold surface.

For the last snapshot of the adsorbed protein on gold wall from TIP3P water, we took the following steps. First, all the water molecules were removed from the simulation box. This is an important step to position the second gold wall, which will imitate a Au NP approaching the one with an adsorbed BSA. Since the NP assembly in experiments happens under drying conditions, removing water from the simulation system seems to be justified. Second, a more realistic scenario requires a covalent bond between the sulfur atom of the sulfhydryl group of the reduced cysteine residue in the BSA structure and a neighbouring gold atom of the wall. The parameters for this chemical bond have a length of 2.65 Å and a force constant of 50 kcal mol^−1^ Å^−2^. Third, we positioned the second gold wall parallel to the first one with an adsorbed BSA, maintaining an initial distance between them of approximately 82 Å. Fourth, the adsorbed BSA was equilibrated again for the next 50 ns being in-between two fixed walls.

In the experimental setup, the closest approach between NPs of the unstretched chain was about 1.5 nm, smaller than the diameter of the BSA. Therefore, in the next step, the two walls of the simulation setup had to be pushed together to squeeze the molecular conformation. This was achieved by applying an external force to the mobile gold wall. We applied a force of 100 kcal mol^−1^ Å^−1^ on each gold atom of the mobile wall, with additional optimization geometry runs for BSA every 5–7 ns of simulation time. In total, ~50 ns of simulation time was used to bring the two walls to 1.5 nm. Once again, the BSA conformation in this squeezed state was equilibrated for 50 ns.

Finally, we modelled the stretching of the NP chain by slowly moving the mobile gold wall away from the fixed one, keeping them parallel to each other.

Despite their inherent limitations, the simulations, performed at varying levels of model complexity, convincingly show that during protein adsorption and subsequent squeezing, the BSA’s regular secondary structure first partially and then almost completely vanishes. The results revealed that at a gap size of 1.5 nm between the Au NPs, no α-helices remained intact—they were transformed into coils or, to a lesser extent, into β-turns. BSA proteins, being in the interparticle gap of this size, can be thought of as being glued/adsorbed to/onto the neighbouring NPs. During the subsequent stretching, the protein showed a tendency to partially recover its secondary structure, which was accompanied by a 90° reorientation of its dipole moment in the direction of deformation.

The analysis of the secondary structure was done in visual molecular dynamics by the construction of Ramachandran maps for pristine BSA, BSA in the gap of 1.5 nm between two gold walls, and at distances of 5 and 10 nm between the walls. For the same geometries, the CD spectra of the protein were calculated using online CD spectra calculator PDBMD2CD (ref. ^54^).

### Enzymatic removal of BSA from Au NP assemblies

To remove the BSA corona from the Au NP assemblies, a working solution of Proteinase K was freshly prepared at 100 µg ml^−1^ in 50-mM Tris–HCl buffer (pH 8.0, adjusted with 10% HCl). PDMS-supported Au NP assemblies were uniaxially stretched to 40% strain using a custom-made stretching device, fixed in the strained state, and immediately covered with Proteinase K solution, ensuring complete wetting of the active area. The substrates were then incubated in a preheated oven at 50 °C for 1 h/overnight to allow the enzymatic degradation of surface-bound BSA. After incubation, samples were rinsed three times with Milli-Q water, gently dried under a nitrogen stream and relaxed from the stretching device. The cleaned assemblies were used directly for CD measurements.

### Characterization

Extinction and CD spectra were obtained using an RC2 ellipsometer instrument (J.A. Woollam). To in situ monitor the PCCD under different strains, we mounted the sample on a custom-built stretching device and attached it on the ellipsometer to allow the light beam to pass normally through the sample. AFM images were recorded on a Dimension Series FastScan (Bruker-Nano) with the custom-made stretching stage in the tapping mode with NanoScope 9.7 using stiff cantilevers TESPA (40 N m^−1^, 300 kHz, Tap300, Budget Sensors). TEM images were captured using a ZEISS transmission electron microscope Libra 120. The SERS measurements were collected in an ambient atmosphere with a Renishaw inVia confocal Raman microscope. The sample, mounted on the stretching device, was illuminated with a 785-nm laser line through a ×50 objective, providing a spatial resolution of ~1 μm. Laser power at the sample was set to 1 μW with acquisition times of 1 s. The experiment was repeated in ten different positions of the same sample on stretching. Attenuated total reflectance Fourier transform infrared (ATR-FTIR) measurements were conducted using a Tensor II FTIR spectrometer (Bruker Optics) equipped with a globar source, a mercury cadmium telluride detector and a dedicated mirror setup for ATR-FTIR analysis (OptiSpec). The setup included a holder for a trapezoidal silicon internal reflection element. A piece of PDMS substrate with deposited BSA-coated Au NPs was pressed onto the Si internal reflection element. Sample intensity spectra (I_S_) were recorded by averaging 100 scans at a spectral resolution of 2 cm^−1^ (zero filling factor of 2), with the uncoated Si internal reflection element serving as the reference intensity (I_R_). ATR-FTIR spectra were calculated as *A* = −log[I_S_/I_R_] and further processed using OPUS software (v. 7.0, Bruker Optics).

#### COMSOL modelling

The simulations of CD are based on COMSOL Multiphysics and Maxwell’s equations. The chiro-optical properties of the biomedia in our model enter the computation by imposing Pasteur constitutive relations^44^:

$$\begin{array}{l}{{\bf{D}}}_{\omega }={\varepsilon }_{0}\hat{\varepsilon }{{\bf{E}}}_{\omega }+{\rm{i}}{\hat{\xi }}_{{\rm{c}}}{{\bf{H}}}_{\omega }/{{\rm{c}}}_{0},\\ {{\bf{B}}}_{\omega }=\mu {\mu }_{0}{{\bf{H}}}_{\omega }-{\rm{i}}{\hat{\xi }}_{{\rm{c}}}{{\bf{E}}}_{\omega }/{{\rm{c}}}_{0}.\end{array}$$

The chirality tensor, $\hat{\xi }(r)$, is position dependent and non-zero only inside the shell and neck. Similarly, for the local dielectric constant, we have $\hat{\varepsilon }=\hat{\varepsilon }(r)$. Gold was modelled with the Johnson–Christy tables, and the polymer substrate (PDMS) was described with *n* = 1.4. The details for the chiro-optical tensors are given in Supplementary Note 4.

## Online content

Any methods, additional references, Nature Portfolio reporting summaries, source data, extended data, supplementary information, acknowledgements, peer review information; details of author contributions and competing interests; and statements of data and code availability are available at 10.1038/s41563-026-02586-7.

## Supplementary information

Supplementary Information Supplementary Figs. 1–7, Notes 1–4 and Discussion.

Supplementary Video 1 PEI layer acts as a matrix that transfers strain from the PDMS substrate to the NP chains and subsequently to the BSA molecules between the NPs.

Supplementary Video 2 MD simulation of stretching-induced molecular dipole increase.

## Source data

Source Data Fig. 1 Statistical source data.

Source Data Fig. 2 Statistical source data.

Source Data Fig. 3 Statistical source data.

Source Data Fig. 4 Statistical source data.

Source Data Fig. 5 Statistical source data.

## Figures and Tables

**Extended Data Table 1:**
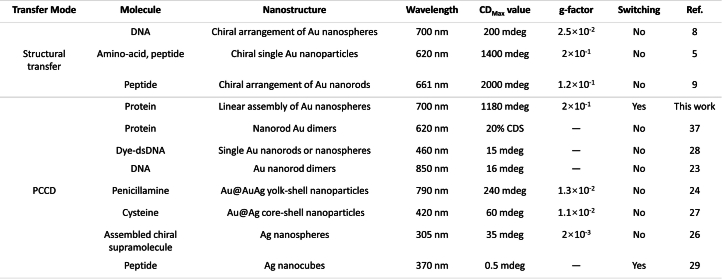
Performance comparison of various PCCD systems comprising chiral molecules and plasmonic nanostructures

With the conventional hotspot-strategy, the g-factor can be increased to the level of 1 × 10^−2^. Our molecule-stretching strategy can break through the bottleneck, elevating the *g*-factor of PCCD to a level of 2 × 10^−1^, enabling efficient induction of chirality from biomolecules^36,37^.

## Data Availability

The data that support the plots in this article and other findings of this study are available via figshare at 10.6084/m9.figshare.28429202 (ref. ^55^). All other data used in this study are available from the corresponding authors upon request. Source data are provided with this paper.
