# Supplementary material for: Efficient and reversible chirality induction between protein and achiral plasmonic assemblies
Source: Nat Mater. 2026 Apr 15;25(7):1230–8. doi: 10.1038/s41563-026-02586-7 (PMC13323087; doi:10.1038/s41563-026-02586-7)
Supplement: Supplementary file 1 — Supplementary Figs. 1–7, Notes 1–4 and Discussion. [file 41563_2026_2586_MOESM1_ESM.pdf]

nature materials

Supplementary information https://doi.org/10.1038/s41563-026-02586-7

# Efficient and reversible chirality induction between protein and achiral plasmonic assemblies

In the format provided by the authors and unedited

# Table of Contents


Supplementary Fig. 1 | TEM characterization of spherical BSA-coated Au NPs. ....................................... 2

Supplementary Fig. 2 | Reference CD measurements to exclude the structural chirality and ensure the pure plasmon coupled circular dichroism (PCCD) in 1D BSA-coated Au NP assembly. .................................... 3

Supplementary Fig. 3 | Sketch of home-made stretching device................................................................. 4

Supplementary Fig. 4 | AFM characterization of the 1D NP assembly array during stretching process....... 5

Supplementary Fig. 5 | The full CD spectrum of 1D BSA-coated Au NP assembly. ................................... 6

Supplementary Fig. 6 | Extinction, CD and g-factor of the 1D BSA-coated Au NP assembly during stretching process. ............................................................................................................................................ 7

Supplementary Fig. 7 | Overstretching of BSA-coated Au NP (70 nm in diameter) assembly. .................... 8

Supplementary Fig. 8 | Excluding the effect of linear dichroism in the stretching-induced PCCD leap by monitoring the Mueller matrix of the assembly under different rotation angles. .......................................... 9

Supplementary Fig. 9 | CD spectra of the BSA@Au nanosphere assembly during stretching taken through conventional CD spectrometry. ......................................................................................................... 10

Supplementary Fig. 10 | Linear assembly of 50 nm NPs. ............................................................................11

Supplementary Fig. 11 | Linear assembly of 90 nm NPs............................................................................. 12

Supplementary Fig. 12 | Monitoring SERS performance during the stretching and relaxing states of the assembly (Phe band at 1080 $cm^{-1}$). ........................................................................................................ 13

Supplementary Fig. 13 | Simulated evolution of BSA secondary structure and CD response under mechanical deformation between AuNPs. ............................................................................................................. 14

Supplementary Fig. 14 | Conformational reorientation of lysine and phenylalanine residues upon stretching. ..................................................................................................................................................... 15

Supplementary Fig. 15 | Simulated strength of the molecular dipole along nanochain direction during continuous stretching................................................................................................................................ 16

Supplementary Fig. 16 | IR spectra of the unstretched and 50% stretched nanochain array. ...................... 17

Supplementary Fig. 17 | Enzymatic removal of BSA from AuNP assemblies without disturbing the plasmonic lattice............................................................................................................................................ 18

Supplementary Note 1: Characterization of circular dichroism (CD) properties using transmission ellipsometry ............................................................................................................................................ 19

Supplementary Note 2: for Cohesive Debonding Simulation ................................................................... 22

Supplementary Note 3: for analysis for Phe and Lys during stretching ..................................................... 24

Supplementary Note 4: COMSOL-computed PCCD spectra.................................................................... 26

Supplementary Video 1. PEI layer to act as a matrix that transfers strain from the PDMS substrate to the NP chains and subsequently to the BSA molecules between the NPs............................................................. 32

Supplementary Video 2. MD simulation of stretching induced molecular dipole increase......................... 32

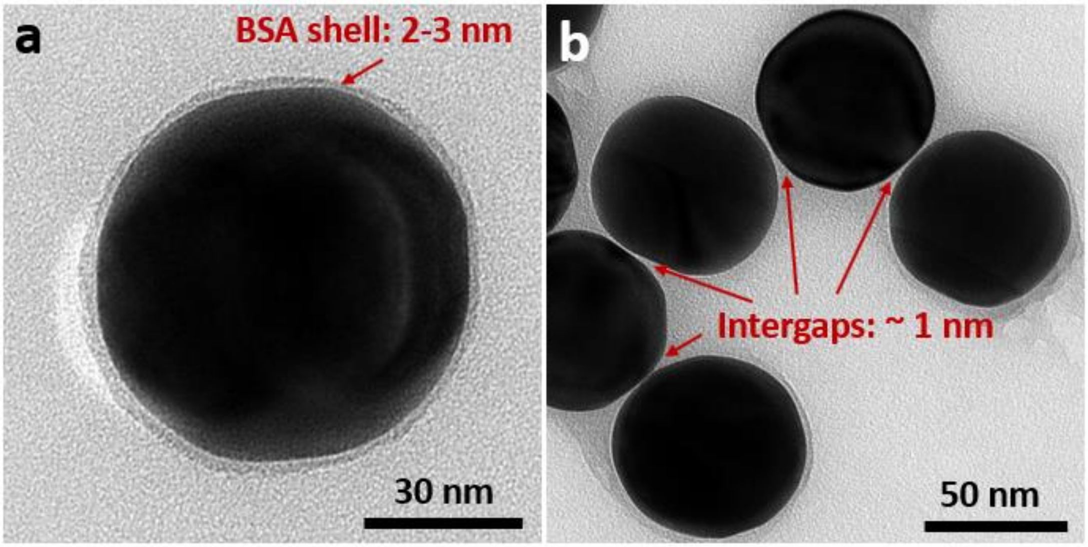


**Supplementary Fig. 1 | TEM characterization of spherical BSA-coated Au NPs. a,** The spherical Au NPs with BSA shell with a thickness of 2-3 nm. **b,** The aggregation of Au NPs. Due to the capillary force, after drying the BSA shells of adjacent NPs were intertwined and pressed. Thus, the size of the intergaps is smaller than the thickness of the isolated Au NP.

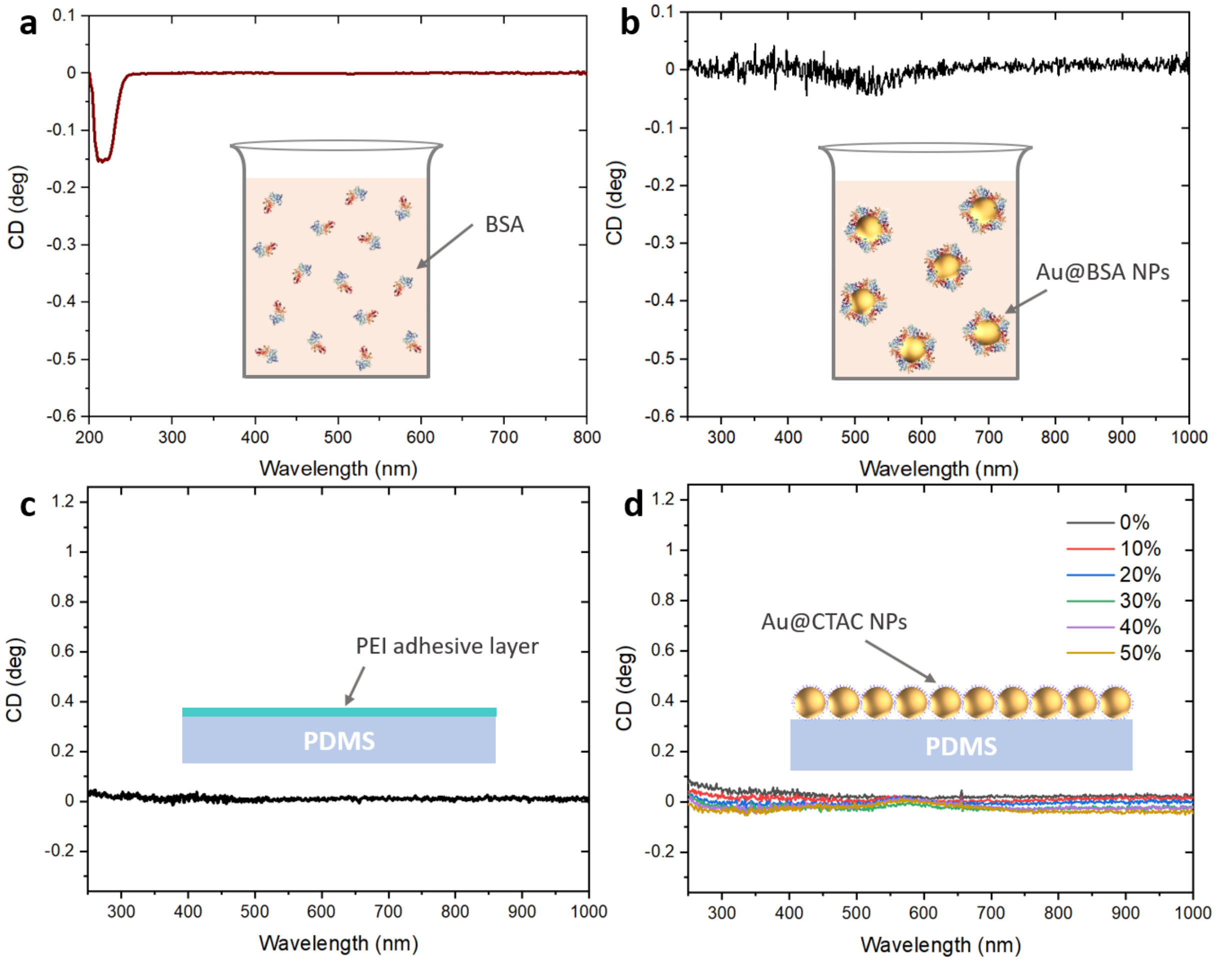


**Supplementary Fig. 2 | Reference CD measurements to exclude the structural chirality and ensure the pure plasmon coupled circular dichroism (PCCD) in 1D BSA-coated Au NP assembly. a,** CD spectrum of 1 mg/mL BSA solution, featuring typical CD peaks of alpha-helix in the UV region. This spectrum was taken from a CD spectrometer with a shorter wavelength range but a higher resolution in UV region compared with the spectra taken from ellipsometer. **b,** CD spectrum of BSA-coated Au NP solution, imparting negligible PCCD and molecular CD of BSA. **c,** CD spectrum of PDMS substrate coated with a PEI adhesion layer, showing no chiral signals. **d,** CD spectrum of 1D CTAC-coated Au NP assembly during stretching, showing its achirality. Because CTAC is not chiral, the PCCD cannot be triggered in this nanocomplex.

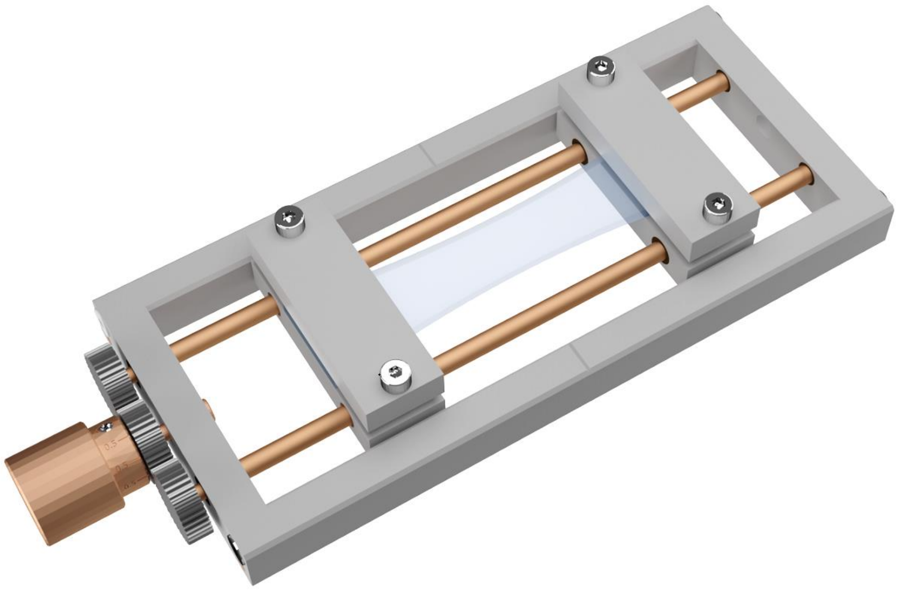

**Supplementary Fig. 3 | Sketch of home-made stretching device.** The PDMS substrate was clapped on the home-made stretching device. By turning the screw, the PDMS substrate will be gradually stretched.

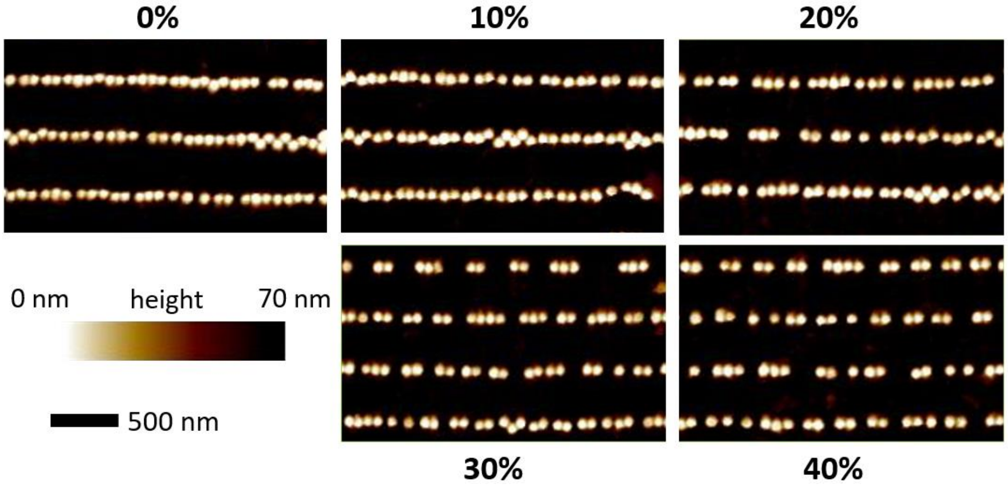


**Supplementary Fig. 4 | AFM characterization of the 1D NP assembly array during stretching process.** When the external strain is gradually applied along the NP chain direction, the NP chains split into oligomers and then to trimers and dimers with 40% strain.

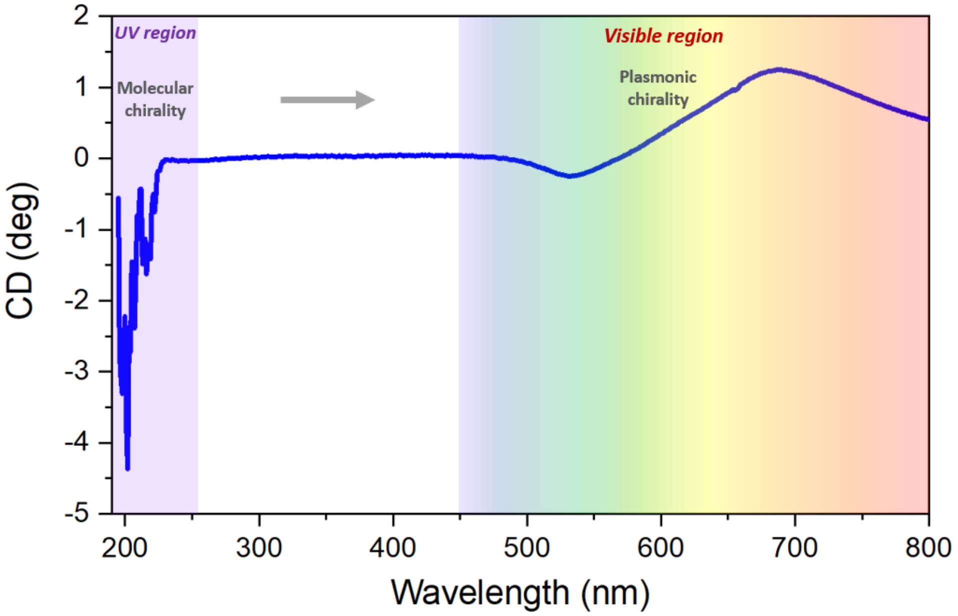


**Supplementary Fig. 5 | The full CD spectrum of 1D BSA-coated Au NP assembly.** In the UV region it shows the typical BSA molecular CD sign in left-handedness with the two characteristic peaks located around 205 nm and 220 nm. While in the visible region, it exhibits a right-handed plasmonic chirality.

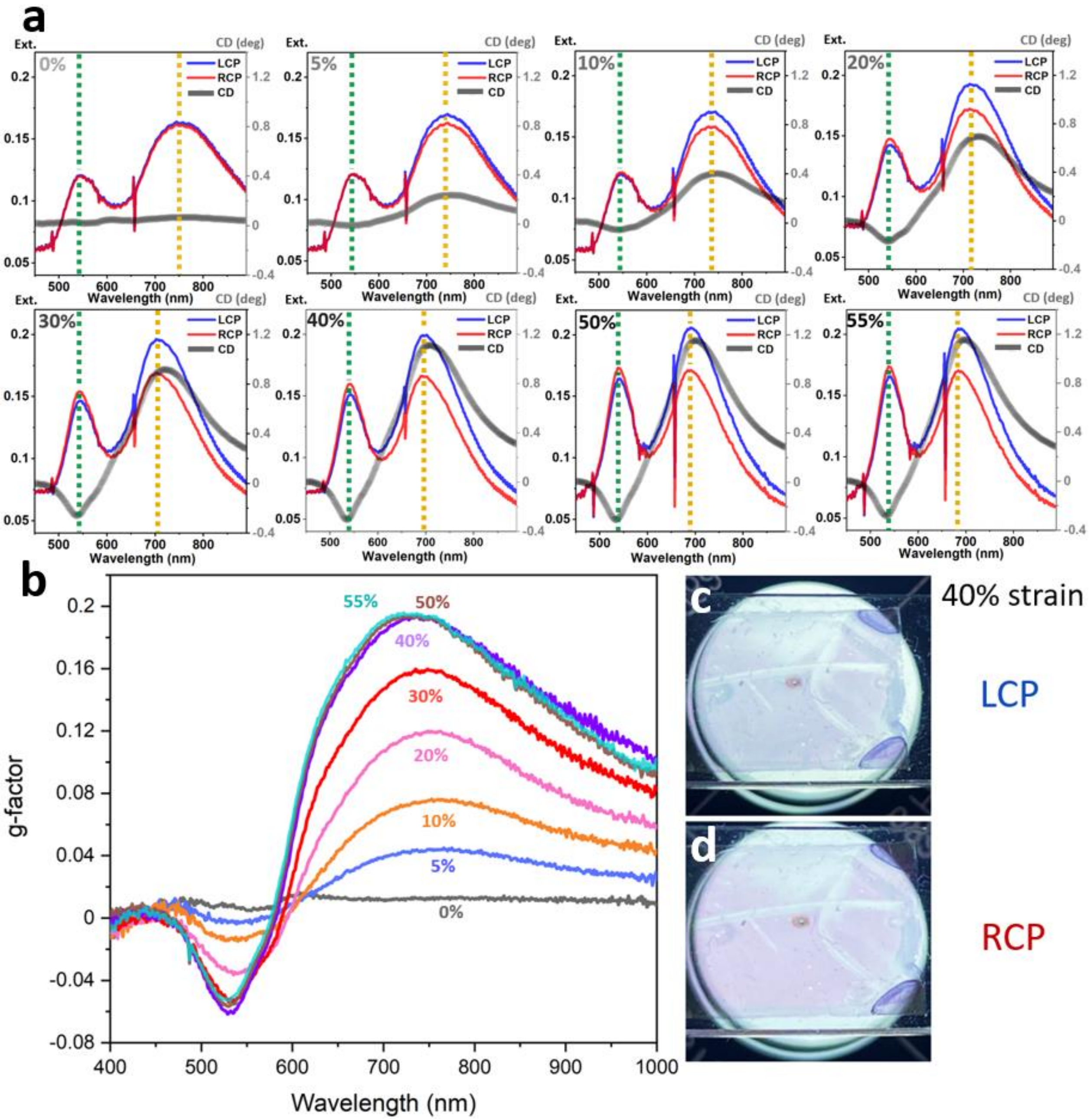


**Supplementary Fig. 6 | Extinction, CD and g-factor of the 1D BSA-coated Au NP assembly during stretching process. a,** Extinction spectra of the BSA-coated Au NP chain assembly (the PDMS substrate baseline subtracted) with increasing strain from 0% to 55% for incident LCP and RCP light. The green dashed lines label the transversal plasmonic mode and the orange dotted lines indicate the longitudinal plasmonic mode. The grey lines represent the corresponding CD spectra for a comparison. **b,** With stretching, the *g*-factor also increases as the CD does, finally reaching a plateau at 50-55% strain with a value of ~0.2. The high *g*-factor allows the assembly for a color difference in transmission when illuminated with LCP and RCP light (**c**, **d**).

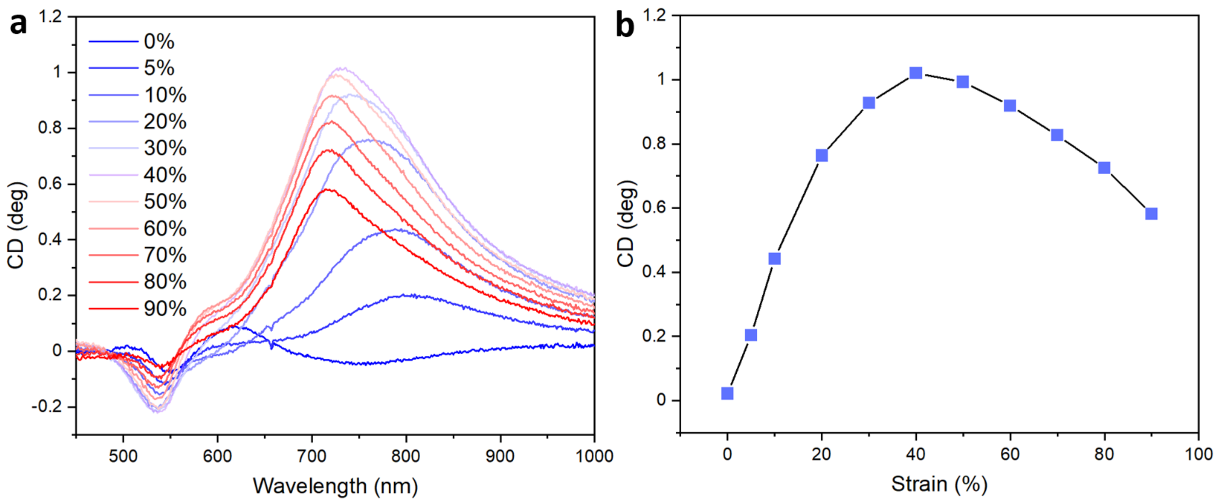


**Supplementary Fig. 7 | Overstretching of BSA-coated Au NP (70 nm in diameter) assembly.** With overstretching, the PCCD decreased slowly together with a gradual blue-shifted longitudinal peak.

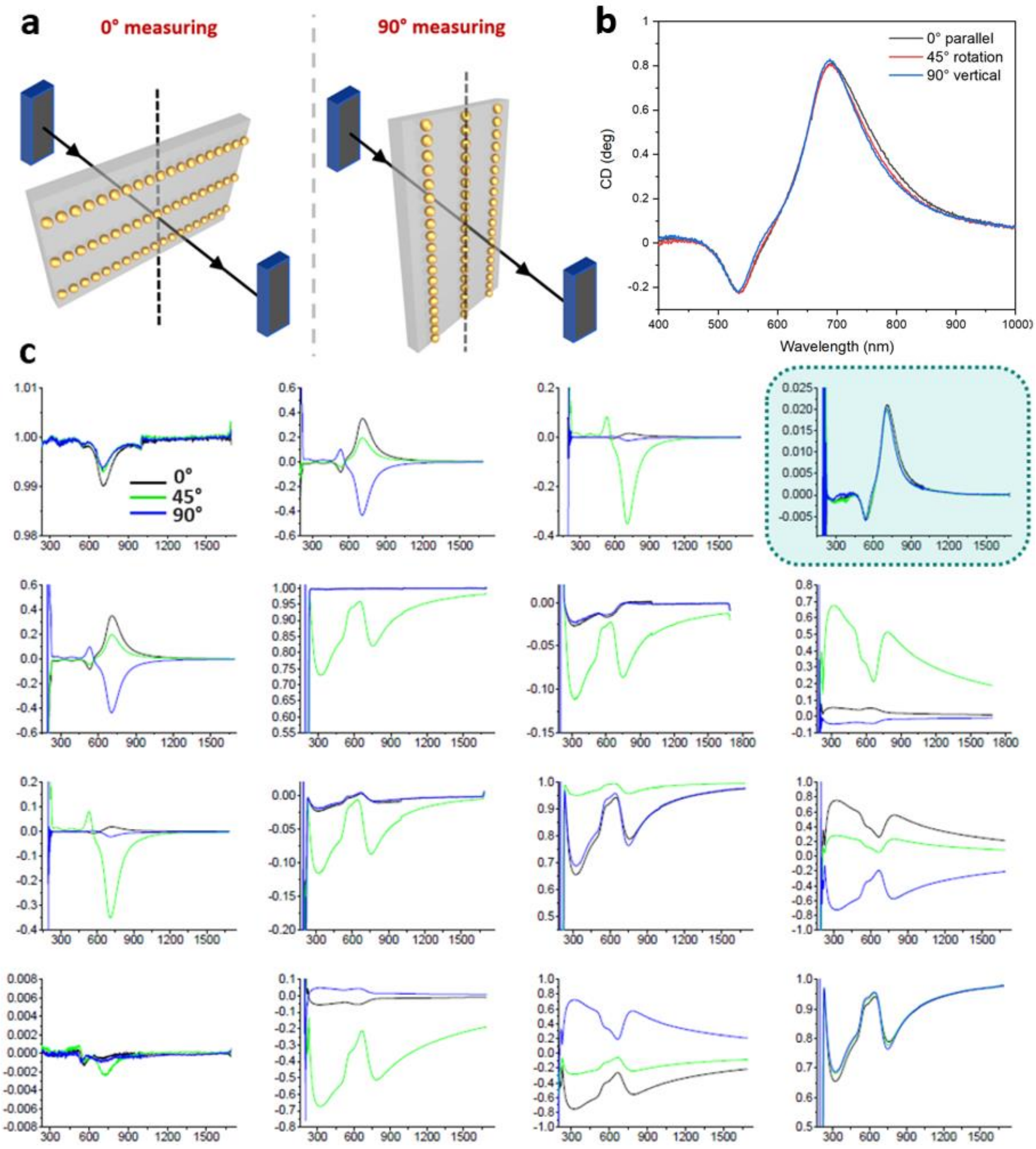


**Supplementary Fig. 8 | Excluding the effect of linear dichroism in the stretching-induced PCCD leap by monitoring the Mueller matrix of the assembly under different rotation angles. a,** Sketch of the rotation experiment through ellipsometer. **b,** Rotating the stretched assembly by 0°, 45° and 90°, the CD values kept always constant. **c,** Full Mueller matrices of the stretched assembly rotated by 0°, 45°, and 90°.

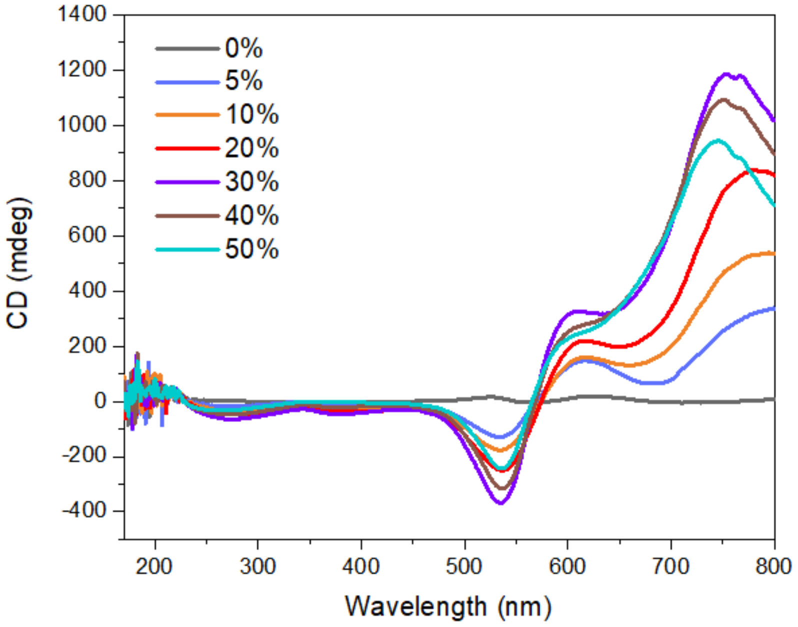


**Supplementary Fig. 9 | CD spectra of the BSA@Au nanosphere assembly during stretching taken through conventional CD spectrometry.** Compared with the CD spectra taken from ellipsometry, nearly the same CD values were also presented here. Due to the limited measuring range of the CD spectrometer, only half of the longitudinal peaks were presented.

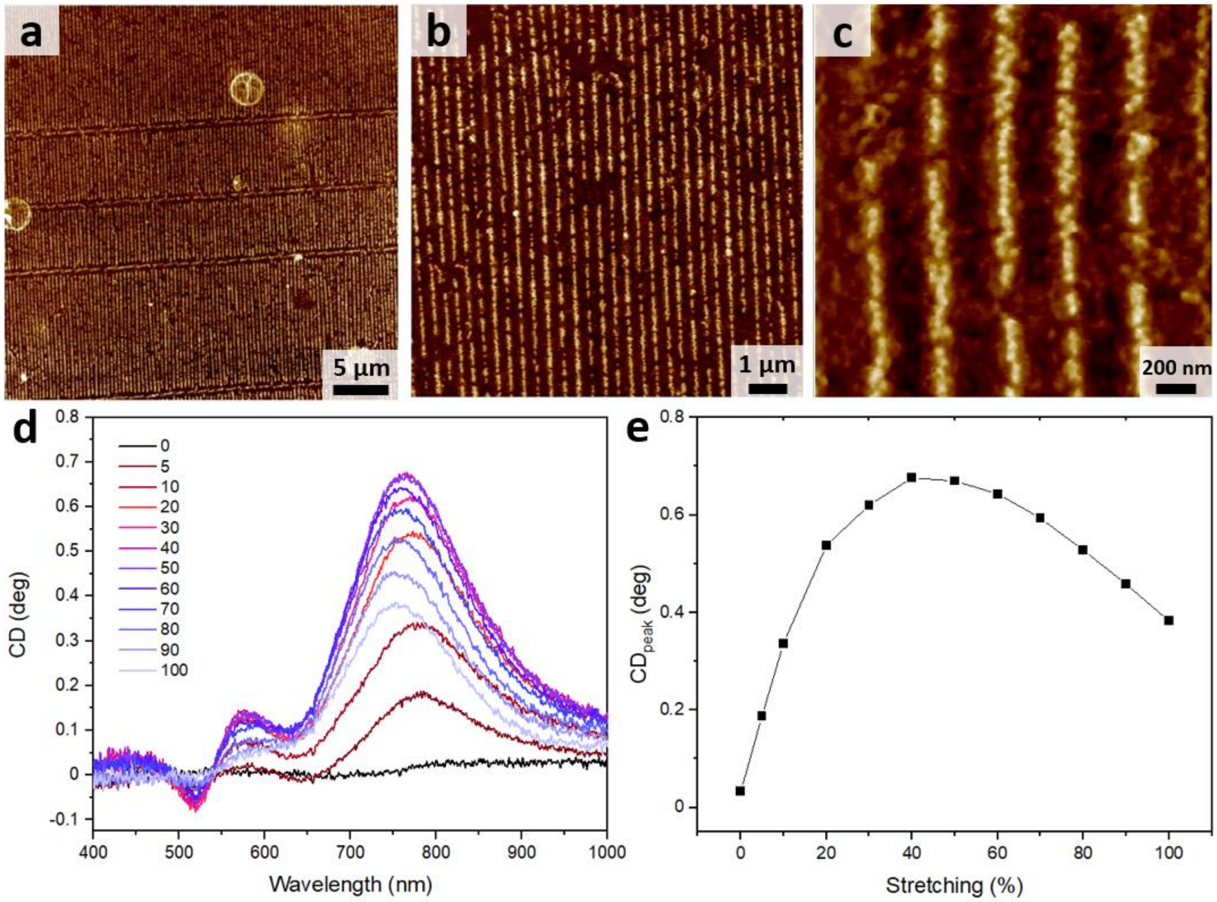


**Supplementary Fig. 10 | Linear assembly of 50 nm NPs. a-c,** AFM images of NP assembly with different magnifications. **d,** Stretching induced CD increase of the NP assembly. **e,** Trend of the CD peak values with continuous stretching.

-

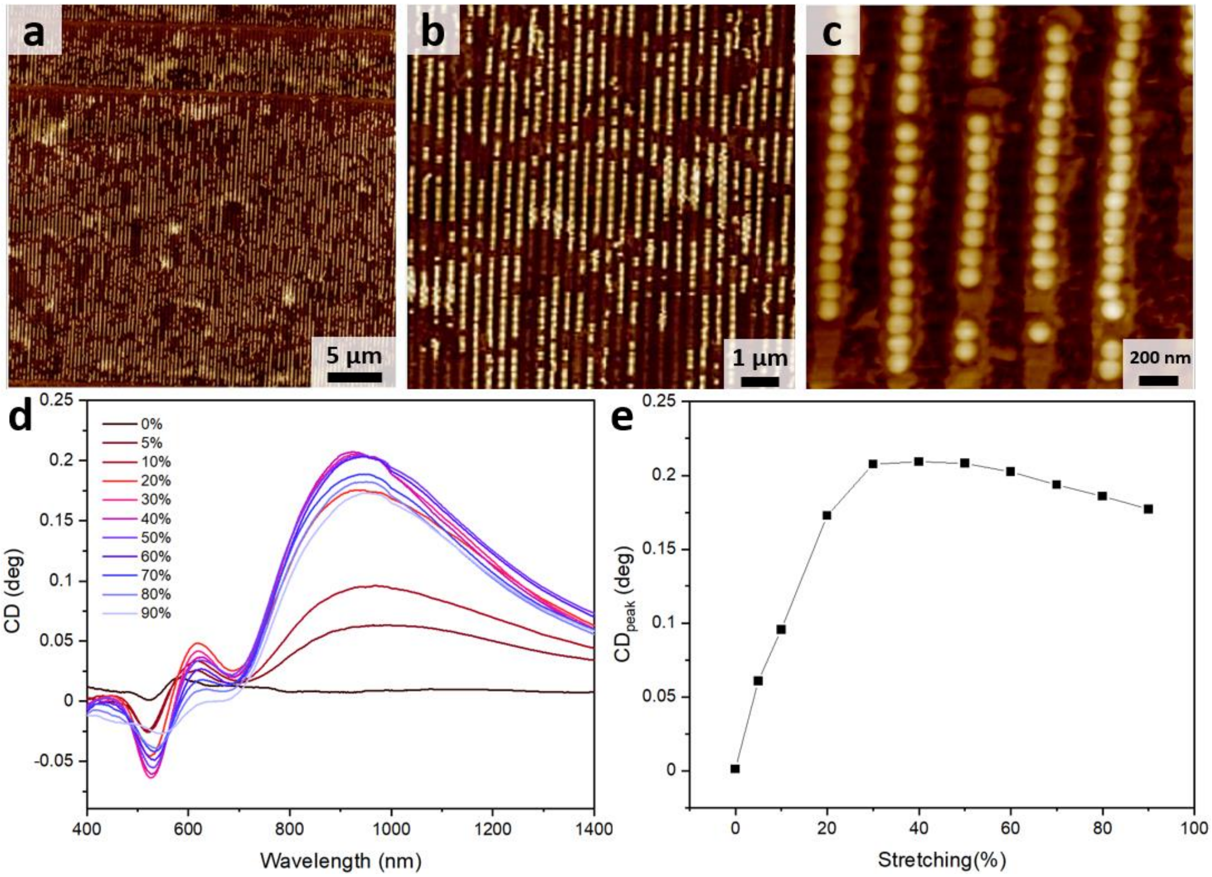


**Supplementary Fig. 11 | Linear assembly of 90 nm NPs. a-c,** AFM images of NP assembly with different magnifications. **d,** Stretching induced CD increase if the NP assembly. **e,** Trend of the CD peak values with continuous stretching.

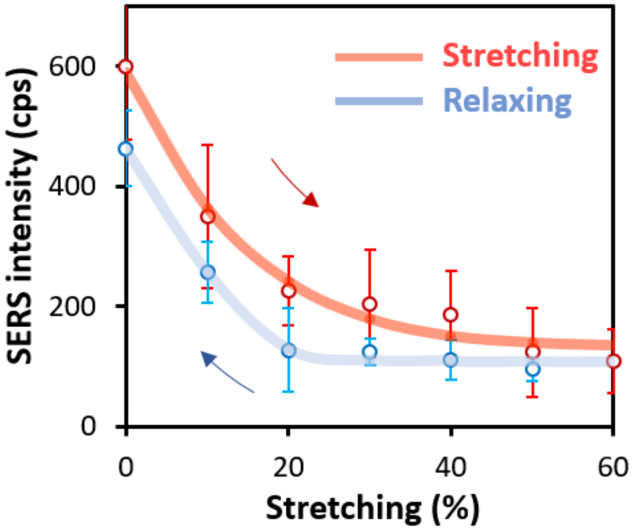


**Supplementary Fig. 12 | Monitoring SERS performance during the stretching and relaxing states of the assembly (Phe band at 1080 cm$^{-1}$).** Upon stretching, SERS signals decrease as nanogaps between adjacent nanospheres enlarge, while during relaxing the nanogaps narrow and SERS signals are gradually restored. SERS intensity was extracted from the ring C–C stretching band of Phe at 1080 cm$^{-1}$. For each strain value in both stretching and relaxing, data points represent the mean of $n = 10$ technical replicates (SERS acquisitions collected at distinct positions on the same NP-chain assembly/substrate). Error bars indicate ± s.d. across replicates. The experimental unit is the individual protein-coated NP-chain assembly on PDMS.

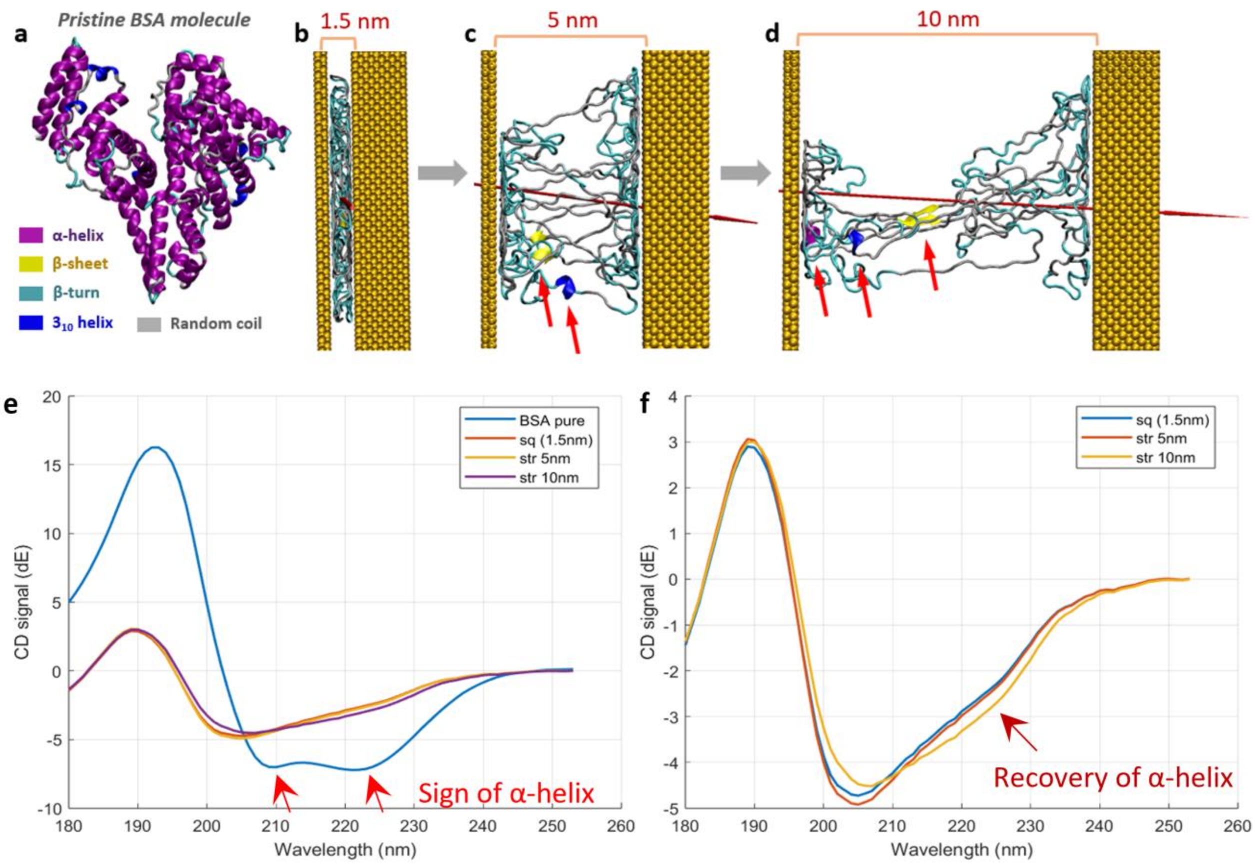


**Supplementary Fig. 13 | Simulated evolution of BSA secondary structure and CD response under mechanical deformation between AuNPs. a-d,** Representative snapshots from molecular simulations showing progressive unfolding and partial refolding of BSA upon compression and stretching within AuNP nanogaps. Compression to a 1.5 nm gap (**b**) disrupts most α-helices, converting most of them into random coil. Subsequent stretching to 5 nm (**c**) promotes a few secondary structures' formation. Further stretching to 10 nm (**d**) further allows local α-helical refolding near the gold surface. **e,** Corresponding simulated CD spectra illustrating loss of the characteristic α-helical negative bands at ~210 and ~222 nm during compression and their partial recovery upon stretching. **f,** Magnified view of (**e**) highlighting the reappearance of the α-helical band near ~222 nm at a 10 nm gap, consistent with localized refolding observed in (**d**).

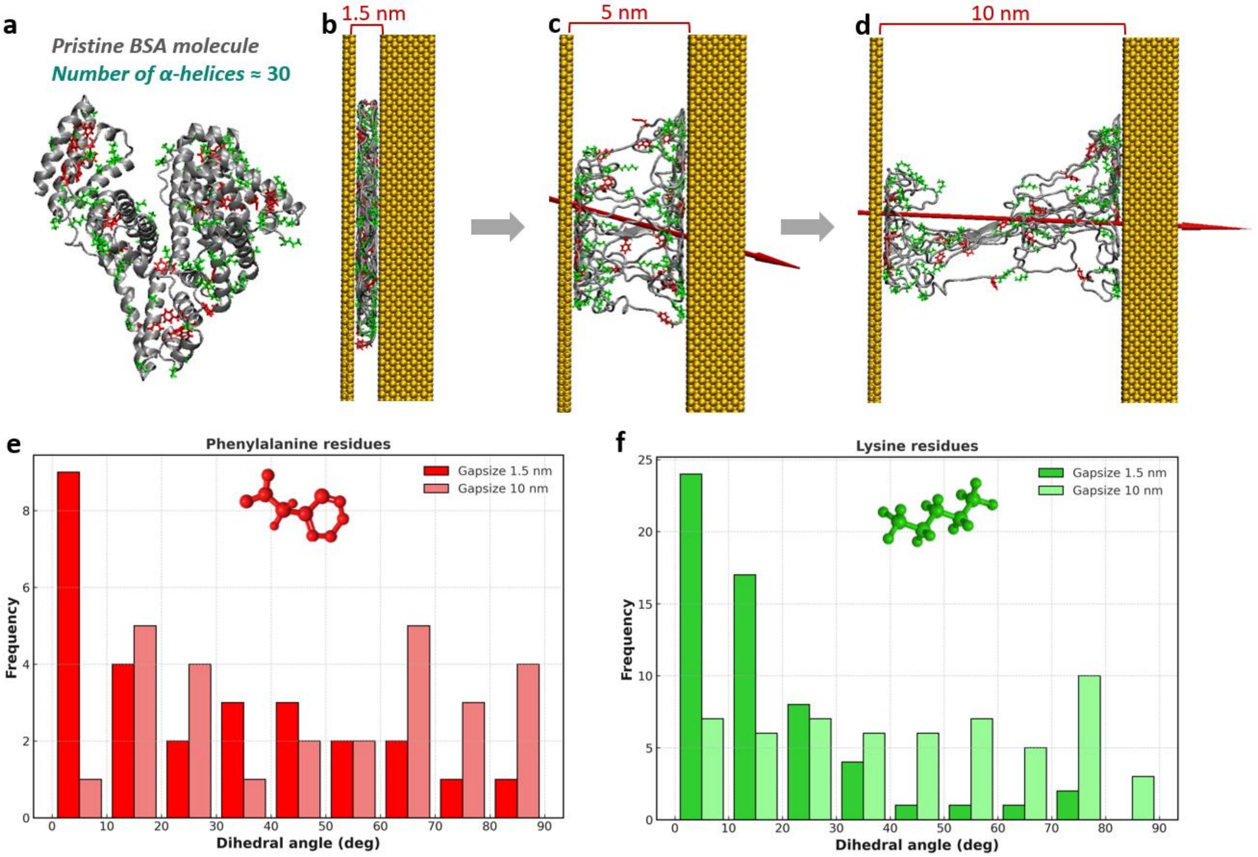


**Supplementary Fig. 14 | Conformational reorientation of lysine and phenylalanine residues upon stretching. a-d,** Structural snapshots of the BSA molecule confined between gold surfaces, illustrating the reorientation of lysine (green) and phenylalanine (red) residues as the gap size increases from 1.5 nm to 10 nm. Approximately 30 α-helices are identified in the native state. Stretching results in partial unfolding and loss of direct residue–gold interactions. **e,f,** Corresponding distributions of dihedral angles for phenylalanine (e) and lysine (f) residues before (1.5 nm) and after stretching (10 nm). The broadening and shift toward larger angles after stretching indicate residue reorientation consistent with protein-level realignment from parallel to more normal orientations relative to the gold surface.

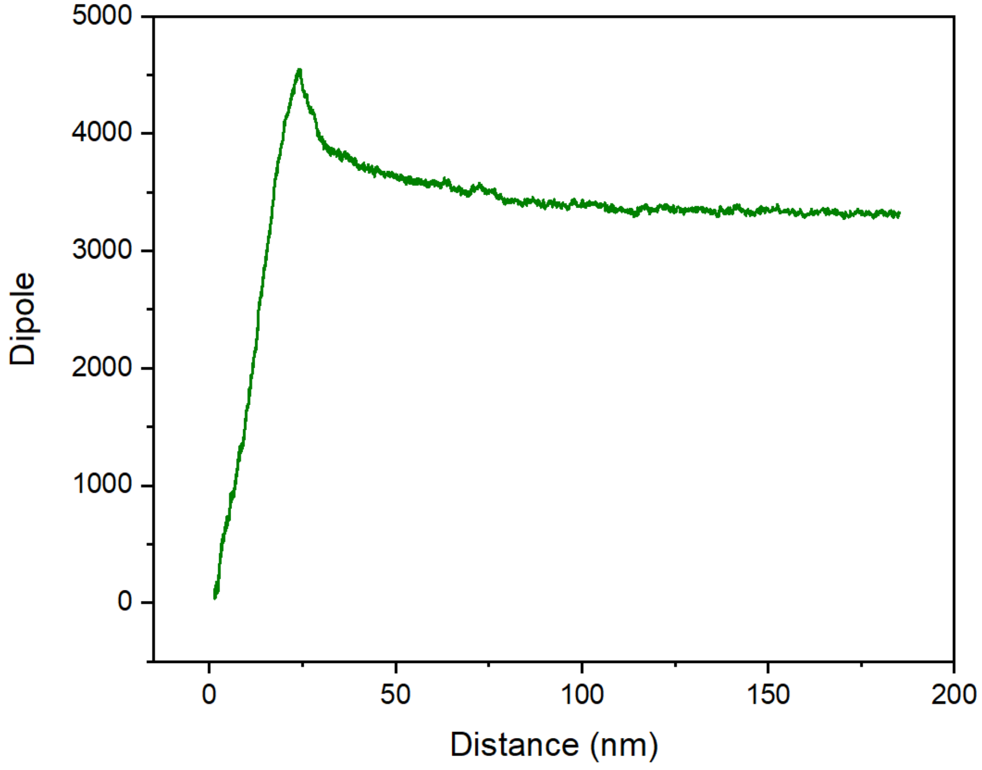


**Supplementary Fig. 15 | Simulated strength of the molecular dipole along nanochain direction during continuous stretching.**

.

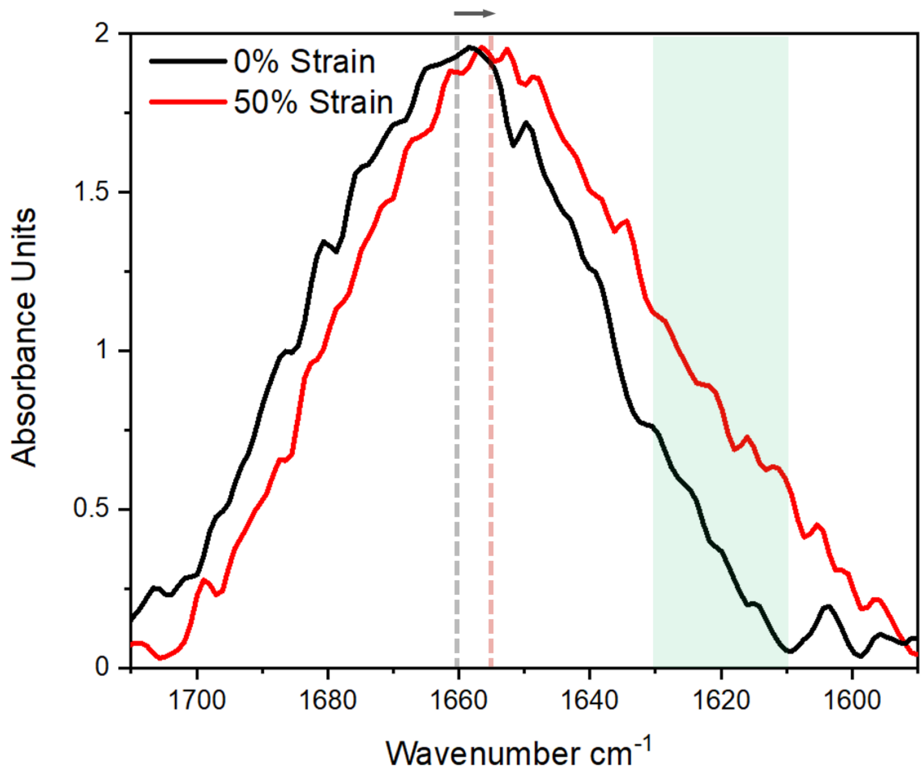


**Supplementary Fig. 16 | IR spectra of the unstretched and 50% stretched nanochain array.**

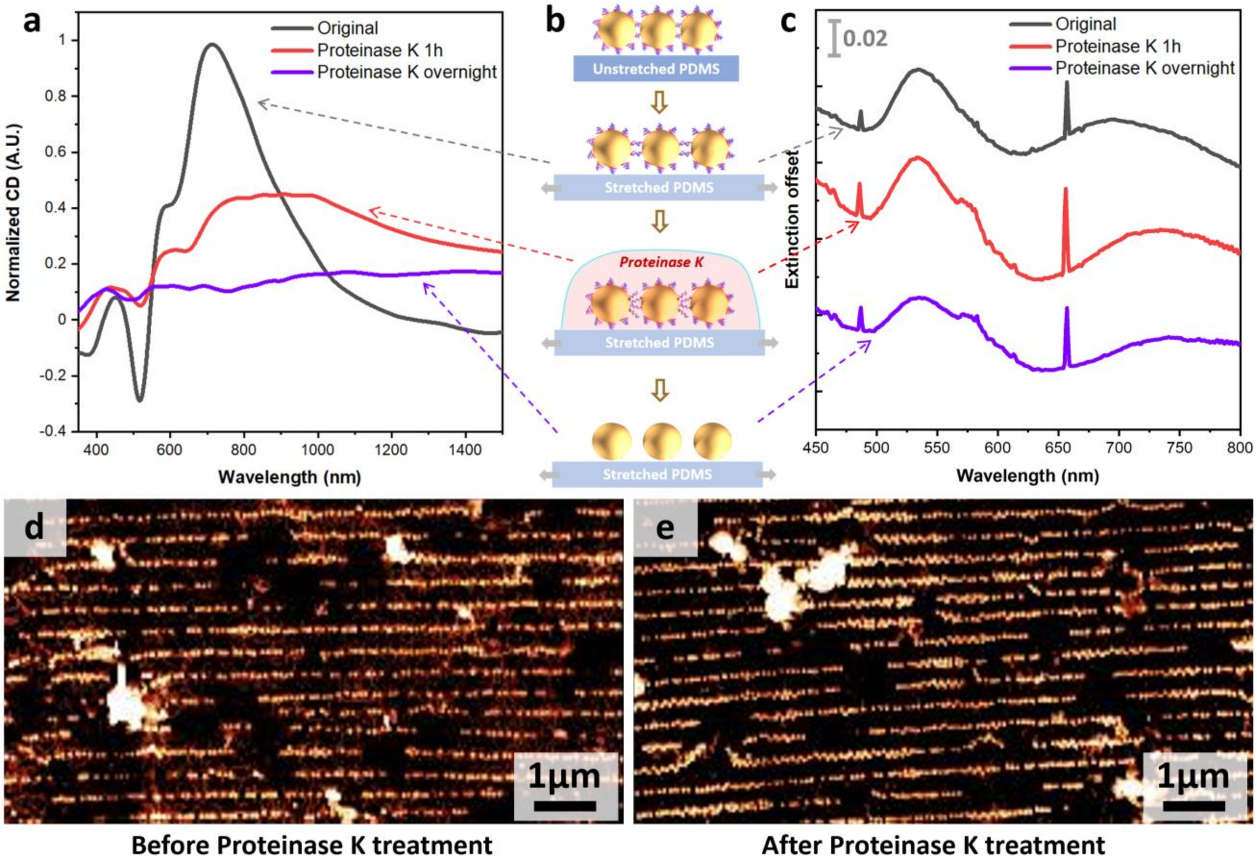


**Supplementary Fig. 17 | Enzymatic removal of BSA from AuNP assemblies without disturbing the plasmonic lattice. a,** CD spectra of stretched BSA-coated AuNP assemblies on PDMS before and after Proteinase K treatment. The original sample (black curve) exhibits strong PCCD in the visible to near-infrared region. Upon incubation with Proteinase K solution for 1 h at 50 °C (red curve), the PCCD signal decreases obviously. After overnight treatment under the same conditions (purple curve), the PCCD is almost completely eliminated, indicating efficient enzymatic degradation of the BSA corona. **b,** Schematic illustration of the BSA-coated AuNP assemblies on stretched PDMS and the enzymatic removal of the BSA shell by Proteinase K. **c,** Extinction spectra (vertically offset for clarity) of the same samples as in **a.** The plasmonic extinction features characteristic of the AuNP lattice are preserved after Proteinase K treatment, despite minor variations in peak position and intensity. This indicates that the nanoparticle assemblies remain largely intact and that the loss of PCCD originates from removal of the BSA corona rather than disruption of the plasmonic lattice. **d,e,** AFM height images of the AuNP assemblies on PDMS before (**d**) and after (**e**) Proteinase K treatment (scale bars, 1 μm), confirming that the long-range order of the nanoparticle arrays is preserved and that the loss of PCCD originates from removal of the BSA shell rather than disruption of the plasmonic lattice.

**Supplementary Note 1: Characterization of circular dichroism (CD) properties using transmission ellipsometry**

CD is typically assessed using CD spectrometers; however, an alternative methodology involves utilizing Mueller matrices (MM) derived from transmission ellipsometry experiments[1]. Both of these strategies were employed in the current study. The transmission ellipsometry experiments were executed using an RC2 spectroscopic ellipsometer (J. A. Woollam), employing a 4×4 MM in transmission mode across the wavelength spectrum of 180-1800 nm.

The polarization response of an optical device or system can be elucidated by a MM, denoted as M. This matrix operates on input polarization states $\boldsymbol{S_{in}} = (S_0, S_1, S_2, S_3)$ and yields corresponding output polarization states $\boldsymbol{S_{out}} = (S_0', S_1', S_2', S_3')$:

$$\begin{bmatrix} S_{0'} \\ S_{1'} \\ S_{2'} \\ S_{3'} \end{bmatrix}_{\text{out}} = \begin{bmatrix} M_{11} & M_{12} & M_{13} & M_{14} \\ M_{21} & M_{22} & M_{23} & M_{24} \\ M_{31} & M_{32} & M_{33} & M_{34} \\ M_{41} & M_{42} & M_{43} & M_{44} \end{bmatrix} \begin{bmatrix} S_0 \\ S_1 \\ S_2 \\ S_3 \end{bmatrix}_{\text{in}} . \tag{1}$$

Here, $S_i$, $S'_i$ are so-called Stokes parameters, and are defined with the intensity of light with four polarizations:

$$\mathbf{s} = \begin{bmatrix} S_0 \\ S_1 \\ S_2 \\ S_3 \end{bmatrix} = \begin{bmatrix} I_x + I_y \\ I_x - I_y \\ I_{+45^\circ} - I_{-45^\circ} \\ I_R - I_L \end{bmatrix}, \tag{2}$$

where $I_x$, $I_y$, $I_{+45o}$ and $I_{-45o}$ correspond to the intensities of linearly polarized light along the x-axis, y-axis, +45°, and −45° directions, respectively, and $I_R$ and $I_L$ represent the intensities of right and left circular polarized light, respectively.

The normalized Stokes vectors $\hat{\boldsymbol{S}}$ and Muller matrix $\hat{M}$ were derived by dividing $S_0$, $S_1$, $S_2$ and $S_3$ by $S_0$ and $M_{xy}$ by $M_{11}$ as follows:

$$\hat{\mathbf{s}} = \frac{1}{s_0} \begin{bmatrix} S_0 \\ S_1 \\ S_2 \\ S_3 \end{bmatrix} = \begin{bmatrix} 1 \\ \hat{S}_1 \\ \hat{S}_2 \\ \hat{S}_3 \end{bmatrix} \tag{3}$$

$$\mathbf{M} = \frac{1}{M_{11}} * \begin{bmatrix} M_{11} & M_{12} & M_{13} & M_{14} \\ M_{21} & M_{22} & M_{23} & M_{24} \\ M_{31} & M_{32} & M_{33} & M_{34} \\ M_{41} & M_{42} & M_{43} & M_{44} \end{bmatrix} = \begin{bmatrix} 1 & m_{12} & m_{13} & m_{14} \\ m_{21} & m_{22} & m_{23} & m_{24} \\ m_{31} & m_{32} & m_{33} & m_{34} \\ m_{41} & m_{42} & m_{43} & m_{44} \end{bmatrix} \tag{4}$$

In this study, we employed Stokes and Mueller calculus to generate CD spectra that correspond to those obtained using the spectrometer.

For the calculation of the CD spectra, we determined the transmitted light intensities for left ($I_L$) and right ($I_R$), circularly polarized light. Our approach involved utilizing the normalized Stokes vectors associated with circularly polarized light, which were then multiplied by a normalized Mueller matrix

$$\widehat{\mathrm{M}} \cdot \widehat{\mathrm{s}_{\mathrm{RCP}}} = \widehat{\mathrm{s}_{\mathrm{out}}^{\mathrm{R}}} \tag{5}$$

which yields

$$\begin{pmatrix} 1 & \hat{M}_{12} & \hat{M}_{13} & \hat{M}_{14} \\ \hat{M}_{21} & \hat{M}_{22} & \hat{M}_{23} & \hat{M}_{24} \\ \hat{M}_{31} & \hat{M}_{32} & \hat{M}_{33} & \hat{M}_{34} \\ \hat{M}_{41} & \hat{M}_{42} & \hat{M}_{43} & \hat{M}_{44} \end{pmatrix} \begin{pmatrix} 1 \\ 0 \\ 0 \\ 1 \end{pmatrix} = \begin{pmatrix} 1 + \hat{M}_{14} \\ \hat{M}_{21} + \hat{M}_{24} \\ \hat{M}_{31} + \hat{M}_{34} \\ \hat{M}_{41} + \hat{M}_{44} \end{pmatrix} \tag{6}$$

The first value, $S_0$, within the resultant Stokes vector, $\hat{S}_{out}{}^{R}$, signifies the light intensity in relation to the initial Stokes vector. Consequently, the intensity of a transmitted right circularly polarized light beam can be expressed as:

$$\mathrm{I_R} = \mathrm{I_0^R} \cdot \left(1 + \widehat{\mathrm{M}_{14}}\right). \tag{7}$$

Likewise, the intensity pertaining to left circularly polarized light is determined by the multiplication of a normalized Mueller matrix with the Stokes vector corresponding to left circularly polarized light,

$$\widehat{\mathrm{M}} \cdot \widehat{\mathrm{s}_{\mathrm{LCP}}} = \widehat{\mathrm{s}_{\mathrm{out}}^{\mathrm{L}}} \tag{8}$$

which yielded

$$\begin{pmatrix} 1 & \hat{M}_{12} & \hat{M}_{13} & \hat{M}_{14} \\ \hat{M}_{21} & \hat{M}_{22} & \hat{M}_{23} & \hat{M}_{24} \\ \hat{M}_{31} & \hat{M}_{32} & \hat{M}_{33} & \hat{M}_{34} \\ \hat{M}_{41} & \hat{M}_{42} & \hat{M}_{43} & \hat{M}_{44} \end{pmatrix} \begin{pmatrix} 1 \\ 0 \\ 0 \\ -1 \end{pmatrix} = \begin{pmatrix} 1-\hat{M}_{14} \\ \hat{M}_{21}-\hat{M}_{24} \\ \hat{M}_{31}-\hat{M}_{34} \\ \hat{M}_{41}-\hat{M}_{44} \end{pmatrix} \quad (9)$$

The first value, $S_0$, of the resulting Stokes vector within the resultant Stokes vector, $S_{out}{}^{L}$, signifies the light intensity in relation to the initial Stokes vector. Consequently, the intensity of a transmitted left circularly polarized light beam can be expressed as:

$$\mathrm{I_L} = \mathrm{I_0^L} \cdot \left(1 - \widehat{\mathrm{M_{14}}}\right). \quad (10)$$

By combining Equations 7 and 10, we derived an equation that establishes a connection between normalized Mueller matrices and CD, expressed as:

$$\Delta\mathrm{Ext_{LCP/RCP}} = \log_{10}\left(\frac{\mathrm{I_0^R}\cdot\left(1+\widehat{\mathrm{M_{14}}}\right)}{\mathrm{I_0^L}\cdot\left(1-\widehat{\mathrm{M_{14}}}\right)}\right). \quad (11)$$

Assuming an equivalence in the initial intensity of left and right circularly polarized light ($I_0{}^{R}=I_0{}^{L}$), we obtained

$$\Delta Ext_{LCP/RCP} = \log_{10}\left(\frac{1+\widehat{M_{14}}}{1-\widehat{M_{14}}}\right) \quad (12)$$

or as CD in degrees:

$$\mathrm{CD} = \left(\frac{\ln 10}{4}\right)\left(\frac{180}{\pi}\right)\Delta E\mathrm{xt}_{LCP/RCP}. \quad (13)$$

Moreover, the dissymmetry factor can be calculated as

$$\mathrm{g} = \frac{\mathrm{CD}}{32.982 * \mathrm{Abs}} = \frac{\log\left(\frac{\mathrm{M_{11}}-\mathrm{M_{14}}}{\mathrm{M_{11}}+\mathrm{M_{14}}}\right)}{\log \mathrm{M_{11}}}. \quad (14)$$

The rotation test was also conducted to eliminate the influence of the plasmonic lattice.

**Ref 1**. 1 Querejeta-Fernández, A. *et al.* Circular Dichroism of Chiral Nematic Films of Cellulose Nanocrystals Loaded with Plasmonic Nanoparticles. *ACS Nano* **9**, 10377-10385, doi:10.1021/acsnano.5b04552 (2015).

**Supplementary Note 2: for Cohesive Debonding Simulation**

The Finite Element numerical modeling presented in this study aims to simulate the classical cohesive traction-separation behavior of spherical particles subjected to tensile stretching. To provide a general understanding of the constitutive law, a brief theoretical foundation is introduced herein. We define a term, T to represent the cohesive traction at the debonding surface, which is oriented outward from the surface. In addition, we use the term u to denote the displacement jump between the pair of faces bonded. The directions of T and u are exactly opposite. In this context, we consider a classical traction-separation law, as outlined in Ref 1, with the pseudo-potential functional defined as follows:

$$W_{TS} = W - \frac{|u| + \Delta}{\Delta} \exp\left( -\frac{|u|}{\Delta} \right)$$

where W is a model parameter representing the work or energy of separation, and Δ is another parameter corresponding to the characteristic opening length. The maximum traction, $T_{max}$, can be indirectly calculated using the relation $T_{max} = \frac{W}{\exp(1) \cdot \Delta}$. Through variational derivation, the surface traction can be determined

$$T = -\frac{W}{\Delta^2} \exp\left( -\frac{|u|}{\Delta} \right) |u|$$

The aforementioned traction characterizes the path-independent traction-separation formulation, which models the crack-healing phenomenon during the unloading and reloading processes. Furthermore, an algorithmic approach is proposed to implement a general damage-like formulation that distinguishes between the loading and unloading paths. A representative method involves comparing the current separation to the maximum separation $U_{max}$ attained throughout the entire loading history. The loading case is assumed when the current separation equals the maximum value. In contrast, unloading or reloading is identified when the current separation is smaller than the maximum, at which point the linear traction law is applied. The maximum separation is updated and stored as an internal variable at each loading step. Subsequently, the surface traction is recalculated as

$$T = -\frac{W}{\Delta^2} \exp\left( -\frac{u_{max}}{\Delta} \right) |u|$$

This classical model is implemented in several commercial FE software packages, allowing for direct and straightforward application.

To ensure computational efficiency, we model a limited number of spherical particles in the numerical simulations, treating this as a localized investigation within the broader system. The simulation animation reveals that debonding occurs at the relatively weakened regions, ultimately leading to the formation of clusters where three particles are grouped together and pairs of two particles are also formed occasionally. This behavior closely aligns with the experimental findings in our research.

**Ref 1.** M.J. van den Bosch, P. Schreurs, M. G. D. Geers, On the development of a 3D cohesive zone element in the presence of large deformations, Comput. Mech. 42 (2008) 171–180.

**Supplementary Note 3: for analysis for Phe and Lys during stretching**

*Determination of Torsional Angles between the Gold Surface and Amino Acid Residues*

The torsional angles between the immobile gold surface and the amino acid residues phenylalanine and lysine were determined using an atomistic geometric analysis. The gold surface was defined by three gold atoms residing within the same atomic layer, such that the plane was fully specified by their Cartesian coordinates. For phenylalanine, the orientation of the residue was represented by the plane of the phenyl ring. This plane was constructed using three alternating carbon atoms (C1, C3, and C5) of the aromatic ring. The torsional angle between the gold surface and the phenyl ring was thus evaluated as the dihedral angle between two planes, each defined by three atomic points. In the case of lysine, the angle was defined between the gold surface and the vector connecting the Cα atom and the nitrogen atom of the terminal ammonium group. Accordingly, this parameter corresponds to the angle between a plane and a vector.

All analyses were conducted using the final molecular dynamics snapshots, corresponding to the equilibrated configurations at inter-surface separations of 1.5 nm and at the stretched state where the distance between the gold walls reached 10 nm. The resulting angular distributions were compiled into histograms, as shown in the accompanying figure.

*Orientational Behavior of Phe and Lys Residues of BSA Under Nanoscale Confinement*

The orientational distributions of phenylalanine and lysine residues of BSA with respect to the immobile gold surface were analyzed at the gold-gold inter-surface separations of 1.5 nm and 10 nm, corresponding respectively to the confined and extended states of the system (Supplementary Fig. 14).

For phenylalanine, the dihedral angle was defined between the plane of the phenyl ring and the plane of the gold surface. At a gap of 1.5 nm, the distribution is sharply peaked near small dihedral angles (< 10°), revealing that the aromatic rings preferentially adopt orientations parallel to the gold surface. This alignment is indicative of strong π–metal coupling and interfacial stabilization under confinement, where steric constraints and dispersive interactions favor surface adsorption. Upon increasing the inter-wall separation to 10 nm, the angular distribution becomes broader and more heterogeneous, with multiple local maxima in the 20-80° range. The loss of a dominant orientation reflects reduced interfacial interaction and enhanced conformational entropy as the residue

moves away from the metal interface.

For lysine, the angle was defined between the gold surface and the vector connecting the Cα atom and the nitrogen atom of the terminal ammonium group. At 1.5 nm, the distribution again peaks at low angles, indicating that the lysine side chains are predominantly oriented parallel to the gold surface. This suggests that under strong confinement, the positively charged ammonium groups interact favorably with the polarizable metal interface, promoting an adsorbed, surface-aligned configuration. When the gap is expanded to 10 nm, the orientation becomes nearly uniform across all angles, signifying a loss of surface affinity and a transition to a more isotropic conformational ensemble.

Together, these findings demonstrate distinct yet complementary interfacial behaviors of aromatic and charged residues under nanoscale confinement. While both residues exhibit pronounced alignment with the gold surface at small separations, driven by π-metal and electrostatic interactions respectively, the relaxation of confinement leads to the disappearance of orientational order. This transition highlights the dynamic coupling between molecular conformation, residue chemistry, and confinement scale in determining biomolecular organization at metallic interfaces.

## Supplementary Note 4: COMSOL-computed PCCD spectra

**Structural models.** Supplementary Figures 18a,b depict our theoretical setups constructed using COMSOL Multiphysics. The chirality of the biomedia (the shells and necks within the NP chain) is incorporated into Maxwell's equations through Pasteur constitutive relations: $\mathbf{D}_\omega = \varepsilon_0 \hat{\varepsilon} \mathbf{E}_\omega + i\hat{\xi}_c \mathbf{H}_\omega / c_0$ and $\mathbf{B}_\omega = \mu\mu_0 \mathbf{H}_\omega - i\hat{\xi}_c \mathbf{E}_\omega / c_0$. The corresponding dielectric and chiro-optical tensors in these relations are described below.

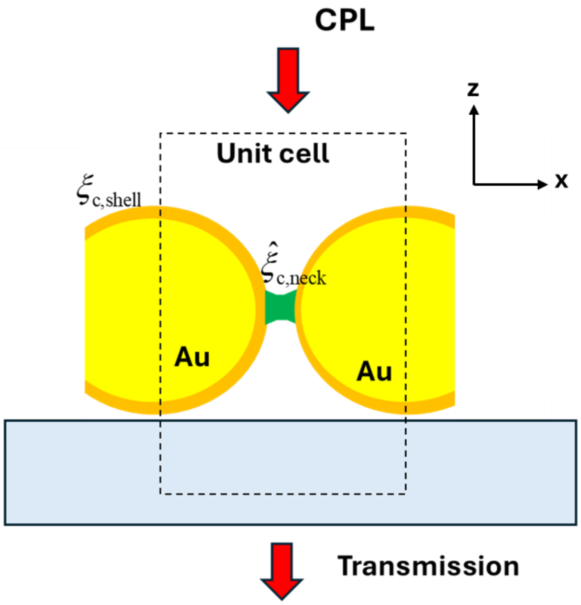


**Supplementary Figure 18a** | The model incorporating local dielectric and chiral properties.

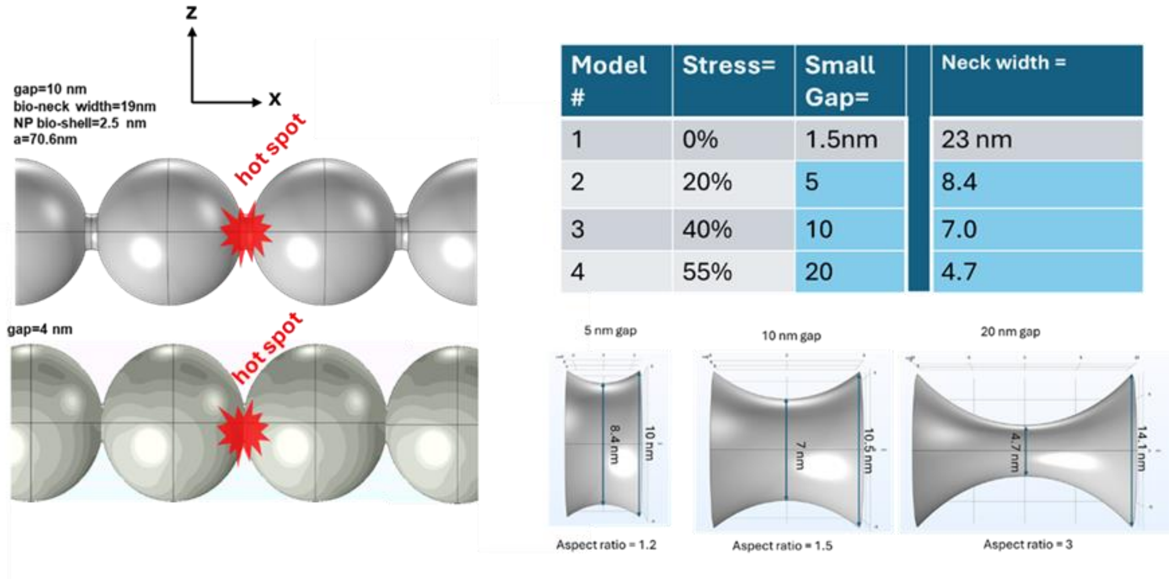


| Model # | Stress= | Small Gap= | Neck width = |
|---|---|---|---|
| 1 | 0% | 1.5nm | 23 nm |
| 2 | 20% | 5 | 8.4 |
| 3 | 40% | 10 | 7.0 |
| 4 | 55% | 20 | 4.7 |

**Supplementary Figure 18b | Geometries of the NP chain and biomolecular necks used in the COMSOL simulations.

The dielectric and chiro-optical dimensionless tensors were modeled as a superposition of a non-stretch isotropic

component and a stretch-induced anisotropic component. The non-stretch term, representing the isotropic response, is expressed as:

$$\hat{\varepsilon}_{non-stretched} = \begin{pmatrix} \varepsilon & 0 & 0 \\ & \varepsilon & 0 \\ & & \varepsilon \end{pmatrix}$$

$$\hat{\xi}_{non-stretched} = \begin{pmatrix} \xi & 0 & 0 \\ & \xi & 0 \\ & & \xi \end{pmatrix}$$

We employ this non-stretched molecular tensor for both the NP shells and the unstretched neck region. For the neck, which represents an anisotropic biomedium, we use the following optical tensors to mimic the behavior of stretched proteins within the interparticle gaps of the chain structures. The tensors corresponding to the stretched medium are given by:

$$\hat{\varepsilon}_{stretched} = \begin{pmatrix} \varepsilon_{xx} & 0 & 0 \\ & 0 & 0 \\ & & 0 \end{pmatrix} = \begin{pmatrix} x_1 \cdot \varepsilon & 0 & 0 \\ & \varepsilon & 0 \\ & & \varepsilon \end{pmatrix}$$

$$\hat{\xi}_{stretched} = \begin{pmatrix} \xi_{zz} & 0 & 0 \\ & 0 & 0 \\ & & 0 \end{pmatrix} = \begin{pmatrix} x_2 \cdot \xi & 0 & 0 \\ & 0 & 0 \\ & & 0 \end{pmatrix}$$

Here, $x_1$ and $x_2$ are the coefficients characterizing the enhancement of dipole moments along the stretching direction, x. The resulting tensors are modeled as a superposition of the non-stretched and stretched components:

$$\hat{\varepsilon}_1 = \frac{\alpha \cdot \hat{\varepsilon}_{non-stretched} + \beta \cdot \hat{\varepsilon}_{stretched}}{\alpha + \beta} = \begin{pmatrix} \frac{\alpha \cdot 1 + \beta \cdot x_1}{\alpha + \beta} & 0 & 0 \\ & 1 & 0 \\ & & 1 \end{pmatrix} \cdot \varepsilon$$

$$\hat{\xi}_1 = \frac{\alpha \cdot \hat{\xi}_{non-stretched} + \beta \cdot \hat{\xi}_{stretched}}{\alpha + \beta} = \begin{pmatrix} \frac{\alpha \cdot 1 + \beta \cdot x_2}{\alpha + \beta} & 0 & 0 \\ & \frac{\alpha \cdot 1 + \beta \cdot 0}{\alpha + \beta} & 0 \\ & & \frac{\alpha \cdot 1 + \beta \cdot 0}{\alpha + \beta} \end{pmatrix} \cdot \xi$$

$$\alpha + \beta = 1$$

The tensors describing the stretched medium exhibit an enhanced dipole moment along the x-direction, corresponding to the stretching axis. The parameter α quantifies the degree of stretching: α = 1 represents the unstretched chiral medium in the neck, while β = 1 corresponds to the fully stretched case. The empirical functions ε and κ are obtained by fitting the calculated extinction and circular dichroism spectra to the experimental chiroptical spectra of a bulk protein solution. The general quantum expressions for ε and κ are

presented below:

$$\varepsilon = \varepsilon_b - \sum_{j=1}^{n} \gamma_j \left( \frac{1}{\hbar\omega - \hbar\omega_{0,j} + i\Gamma_j} - \frac{1}{\hbar\omega + \hbar\omega_{0,j} + i\Gamma_j} \right)$$

$$\xi = \sum_{j=1}^{n} \beta_j \left( \frac{1}{\hbar\omega - \hbar\omega_{0,j} + i\Gamma_j} + \frac{1}{\hbar\omega + \hbar\omega_{0,j} + i\Gamma_j} \right)$$

Here we provide the parameters for non-stretched proteins in solution, using an approximation with n = 3:

$$\hbar\omega_{0,1} = 6.35eV \ \left(195\ nm\right)$$
$$\hbar\omega_{0,1} = 5.9eV \ \left(210\ nm\right)$$
$$\hbar\omega_{0,1} = 5.51eV \ \left(225\ nm\right)$$
$$\gamma_1 = 0.5eV$$
$$\gamma_2 = 0.2eV$$
$$\gamma_3 = 0.2eV$$
$$\Gamma_1 = 0.3\left[eV\right]$$
$$\Gamma_2 = 0.15\left[eV\right]$$
$$\Gamma_3 = 0.15\left[eV\right]$$

$$\text{Normal state: } \begin{pmatrix} \beta_1 = 0.05\left[eV\right] \\ \beta_2 = -0.03125\left[eV\right] \\ \beta_3 = -0.025\left[eV\right] \end{pmatrix}$$

In the stretched state of protein, we enhance the x-dipole and model the chiro-optical constants in this way:

$$\text{Stretched proteins: } \begin{pmatrix} \beta_1 = x_2 \cdot 0.05\left[eV\right] \\ \beta_2 = -x_2 \cdot 0.03125\left[eV\right] \\ \beta_3 = -x_2 \cdot 0.025\left[eV\right] \end{pmatrix}, \quad x_2 = 1, 10, 20$$

Finally, we present below several computations for the local model incorporating biological chirality and plasmonic Au NPs. The extinction spectra for LPL and CPL were calculated, and the circular dichroism (CD) was subsequently derived according to the conventional definitions of extinction and CD:

$$Ext = 1 - T = A + R$$
$$T + A + R = 1$$
$$CD = Ext_{LCP} - Ext_{RCP}$$

where T, R, and A are the transmission, reflection, and absorption coefficients, correspondingly.

**Characterization of the optical selection rules for the T- and L- collective modes:** A chain of nanoparticles supports both transverse (T) and longitudinal (L) plasmonic modes. Supplementary Figure 19a,b demonstrate that T-modes are selectively excited by incident light polarized along the y-axis, while L-modes are excited by x-polarization. The T-mode closely resembles the plasmon resonance of an isolated nanoparticle, whereas the L-mode possesses a strongly collective nature and is observed to be red-shifted, consistent with theoretical expectations.

These figures clearly show that as the gap between nanoparticles decreases, the L-plasmon undergoes a pronounced red-shift, a result of the emergence of a well-defined collective L-mode along the chain. In contrast, the T-plasmon resonance exhibits little spectral shift, reflecting the much weaker interparticle coupling for transverse modes relative to longitudinal ones in a chain geometry.

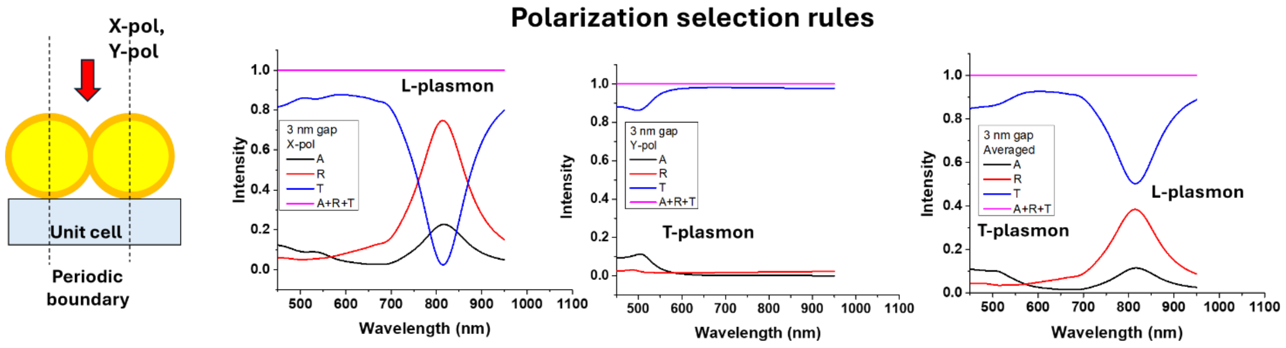


**Supplementary Figure 19a |** Optical responses, T, A, and R, for the NP chain of a 3-nm gap; the panels show the optical configurations of the x- and y-polarizations and the non-polarized case.

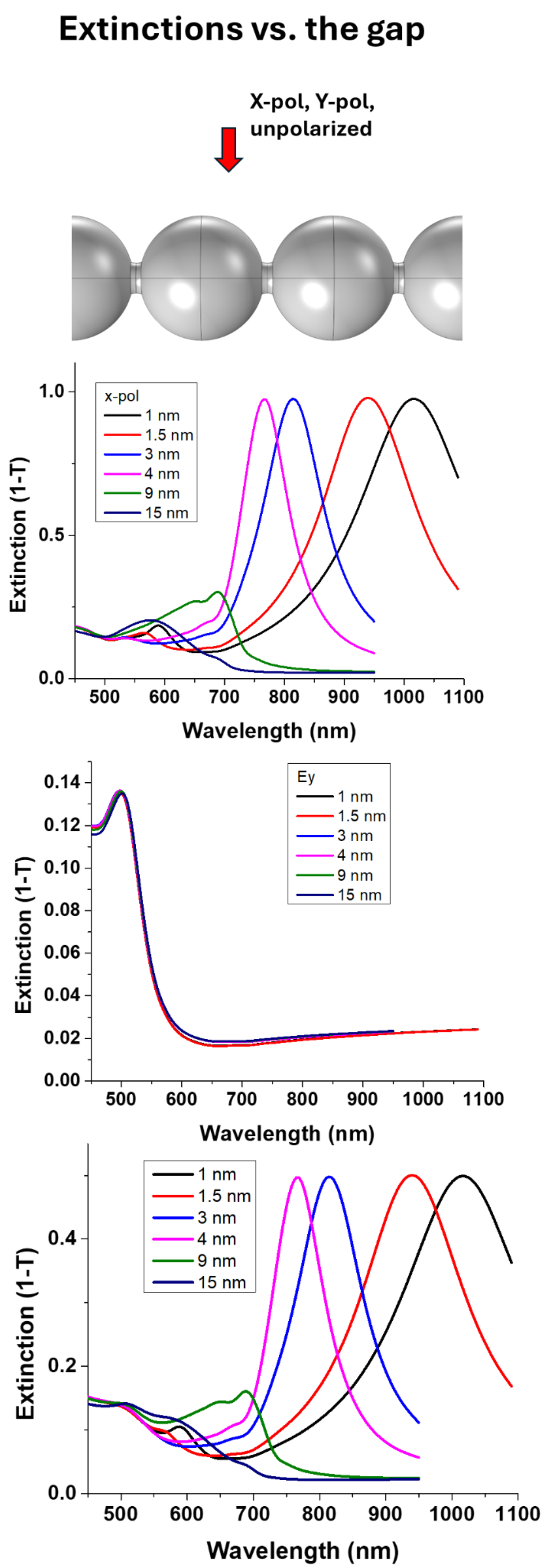


**Supplementary Figure 19b |** We show here the dependence of optical extinction on the interparticle gap in the chain. In these panels, three illumination configurations are shown: x-polarized, y-polarized, and unpolarized incident light. Each configuration reveals distinct extinction features, capturing the polarization-dependent coupling and the evolution of plasmonic modes as the gap varies.

**Chiro-optical effects in the anisotropic and isotropic models of the plasmonic neck:** The main text figures (Figure 4j–l) depict the computed data for the model with an anisotropic chiral tensor, consistent with DFT calculations of dipoles on stretched proteins in the necks. To better understand the robustness of our chiral models, we also computed an isotropic model with an enhanced chiral tensor in the neck. Supplementary 20 shows data for this isotropic model. In these results, the hot-spot volume (i.e., the biomolecular "neck") dominates the CD signal as the parameter $x_2$ increases, reflecting the physical effect of electric field concentration. Accordingly, the total CD strength grows rapidly with increasing chirality in the neck hot-spot region. The bisignate CD shape remains characteristic, with a positive band at the T-plasmon and a smaller negative band at the L-plasmon for the optical configuration with k ∥ −z.

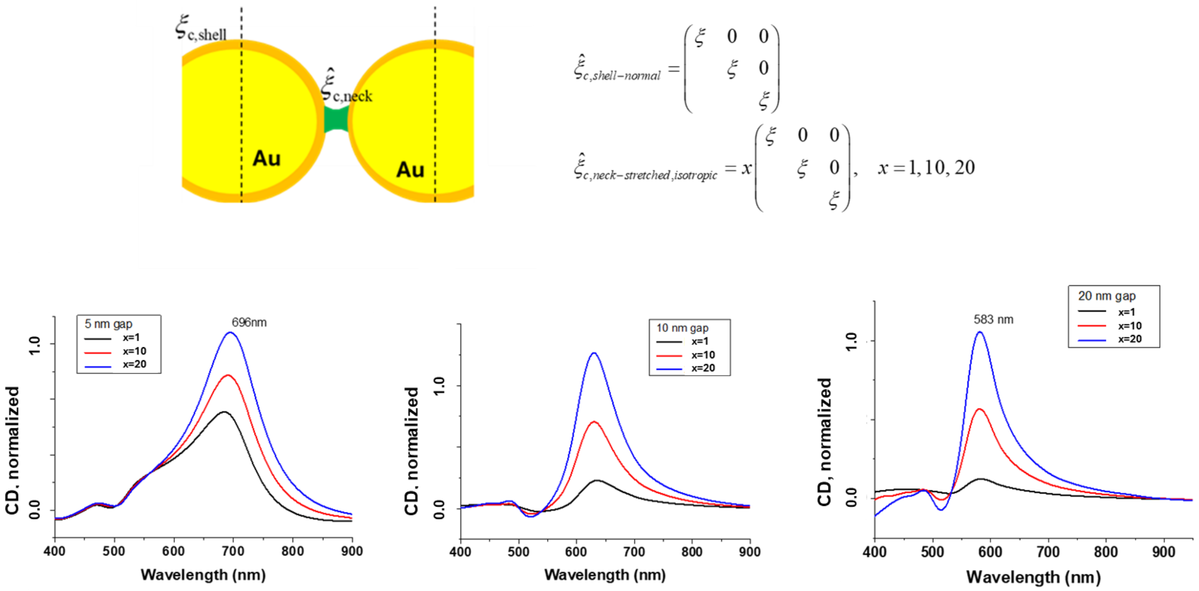


**Supplementary Figure 20 |** COMSOL-computed CD spectra in normalized units for the chains (5, 10, and 20-nm gaps) under the stretching effect with the isotropic chiro-optical tensors.

**Supplementary Video 1.** PEI layer to act as a matrix that transfers strain from the PDMS substrate to the NP chains and subsequently to the BSA molecules between the NPs.

**Supplementary Video 2.** MD simulation of stretching induced molecular dipole increase.
